# Dual‐Stage Microneedle Patch With Lipid Droplet‐Mediated ‘Capture‐Kill’ and Sustained Release for Enhanced Wound Healing

**DOI:** 10.1002/advs.75589

**Published:** 2026-05-06

**Authors:** Peng Jiang, Meijun Jiang, Jiapeng Lei, Wen Zhang, Huiyuan Yang, Xueyang Wang, Siyu Zhang, Qiongfang Ruan, Shuhua Liu, Wei Li

**Affiliations:** ^1^ Department of Burns Tongren Hospital of Wuhan University (Wuhan Third Hospital) School of Pharmaceutical Sciences Wuhan University Wuhan P. R. China; ^2^ Taikang Center for Life and Medical Sciences Wuhan University Wuhan P. R. China

**Keywords:** bacteria targeting, infected wound healing, lipid droplets, microneedles, sonodynamic therapy

## Abstract

Chronic wound infections, particularly those complicated by antibiotic‐resistant bacteria and biofilm formation, present persistent clinical challenges due to limited drug penetration and unresolved inflammation. Here, we report a lipid‐droplet‐engineered, gallium‐loaded sonodynamic therapeutic platform (LDTG) incorporated into a bilayer microneedle (MN) patch co‐delivering all‐trans retinoic acid (ATRA), termed LDTG A‐MNs, for synergistic antibacterial therapy and accelerated wound repair. The engineered LDTG exhibit strong bacteria‐targeting capability by mimicking host lipid droplets and efficiently generate reactive oxygen species (ROS) under ultrasound irradiation to achieve potent sonodynamic bactericidal activity. The bilayer MN design enables rapid release of LDTG for targeted bacterial capture, followed by sustained ATRA release to modulate macrophage polarization and promote tissue regeneration. In vitro, LDTG A‐MNs demonstrate robust antibacterial and biofilm‐disrupting effects, excellent biocompatibility, and enhanced M2‐type macrophage polarization. In vivo, LDTG A‐MNs combined with ultrasound effectively eradicate bacterial infection, suppress excessive inflammation, improve collagen deposition, enhance angiogenesis, and significantly accelerate wound closure in infected murine skin wounds. This work presents a minimally invasive and infection‐responsive MN system that integrates pathogen targeting, sonodynamic antibacterial therapy, and immune‐regulated tissue repair, offering a promising therapeutic strategy for the management of complex and refractory infected wounds.

## Graphical abstract

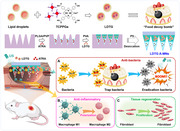

## 1 Introduction

Skin wound infections, particularly those caused by drug‐resistant bacteria, remain a major challenge in clinical wound management [1]. Pathogenic microorganisms such as *Staphylococcus aureus* (*S. aureus*) and *Pseudomonas aeruginosa* (*P. aeruginosa*) can rapidly proliferate at wound sites, leading to persistent inflammation, extensive tissue destruction, and delayed healing [2]. Moreover, these bacteria readily form biofilms, which are complex extracellular matrix structures and act as protective barriers, thereby greatly reducing antibiotic penetration and efficacy [3]. As a result, conventional antibiotic‐based therapies often fail to eradicate bacterial infections and may further contribute to antimicrobial resistance, highlighting the urgent need for alternative therapeutic strategies.

Reactive oxygen species (ROS)‐based antibacterial strategies, distinguished by their broad‐spectrum efficacy and minimal propensity for inducing resistance, have emerged as promising non‐antibiotic approaches for the treatment of infected chronic wounds. For example, wearable ozone‐generating systems have been developed to enable localized and controllable ROS exposure for antibacterial therapy, demonstrating promising efficacy in infected dermal wound models [4, 5, 6]. However, such strategies rely on exogenously supplied ROS, which may suffer from limited tissue penetration, insufficient targeting specificity, and potential off‐target oxidative damage to surrounding healthy tissues. In contrast, sonodynamic therapy (SDT), which leverages ultrasound (US) to activate sonosensitizers for in situ generation of ROS, has gained increasing attention as an effective strategy for treating deep‐seated bacterial infections [7, 8, 9], since SDT offers deeper tissue penetration, controllable spatiotemporal activation, and reduced phototoxicity [10]. For example, tetrakis (4‐carboxyphenyl) porphyrin (TCPP) acts as a high‐efficiency sonosensitizer by transforming acoustic energy into chemical energy to generate highly toxic ROS under US irradiation. These ROS induce oxidative damage to bacterial membranes and biofilm structures, thereby enabling effective antibacterial therapy. Consequently, a variety of SDT delivery platforms including hydrogel dressings [11, 12], microbubbles [13, 14], and engineered nanoparticles [15, 16], have been developed to enhance antibacterial performance. However, their therapeutic efficacy remains limited by insufficient bacterial targeting, inadequate local accumulation, and suboptimal stability in complex wound environments.

To address these challenges, biomimetic delivery systems have gained increasing attention. Naturally derived biomaterials such as cells [17], membranes, and cell‐derived vesicles [18], exhibit excellent biocompatibility and intrinsic biological functionality [19]. Among them, lipid droplets (LDs), intracellular organelles composed of a phospholipid monolayer surrounding a neutral lipid core [20], are abundant in eukaryotic cells and exist at both nano‐ and microscale dimensions [21]. Beyond their canonical roles in lipid storage and metabolic buffering [22, 23], LDs serve as a nutrient source for pathogenic bacteria, which actively recruit and interact with LDs during infection to exploit their lipid content [24]. This natural affinity enables LDs to function as “capture” substrates for pathogens [24, 25], positioning them as attractive vehicles for targeted antimicrobial delivery [26]. Moreover, LDs can be reliably isolated via simple differential centrifugation, supporting their feasibility for translational applications [27, 28]. Nevertheless, the instability of free LD formulations and limited retention at wound sites restrict their therapeutic utility.

Microneedles (MNs) represent a powerful transdermal delivery platform widely applied in wound therapy [29, 30]. With needle lengths ranging from tens to hundreds of micrometers, MNs can efficiently penetrate the epidermis and even disrupt thick bacterial biofilms, enabling precise delivery of therapeutic agents directly into lesion sites [31, 32]. Recent advances have expanded MN functionality beyond passive delivery to include active antibacterial and microenvironment‐modulating strategies, such as ROS‐generating and oxygen‐releasing systems. For instance, degradable MNs have been engineered to enhance bacterial inactivation and accelerate wound healing through localized therapeutic delivery [33]. Additionally, flexible MN patches capable of oxygen generation have demonstrated efficacy in alleviating hypoxia and eradicating biofilms in chronic wounds [34]. These studies highlight the potential of MN platforms for treating infection‐associated wounds through localized biochemical modulation. Furthermore, innovations in MN architecture—including bilayer [35], core‐shell [36], and modular strategies [37]—provide multifunctional and programmable release profiles that better address the dynamic requirements of wound healing. Although SDT and MN systems have been explored individually or in combination for infection treatment [13, 38, 39, 40], critical challenges remain, including the biosafety of inorganic nanomaterials, insufficient bacterial targeting, and inadequate modulation of inflammatory responses.

**SCHEME 1:**
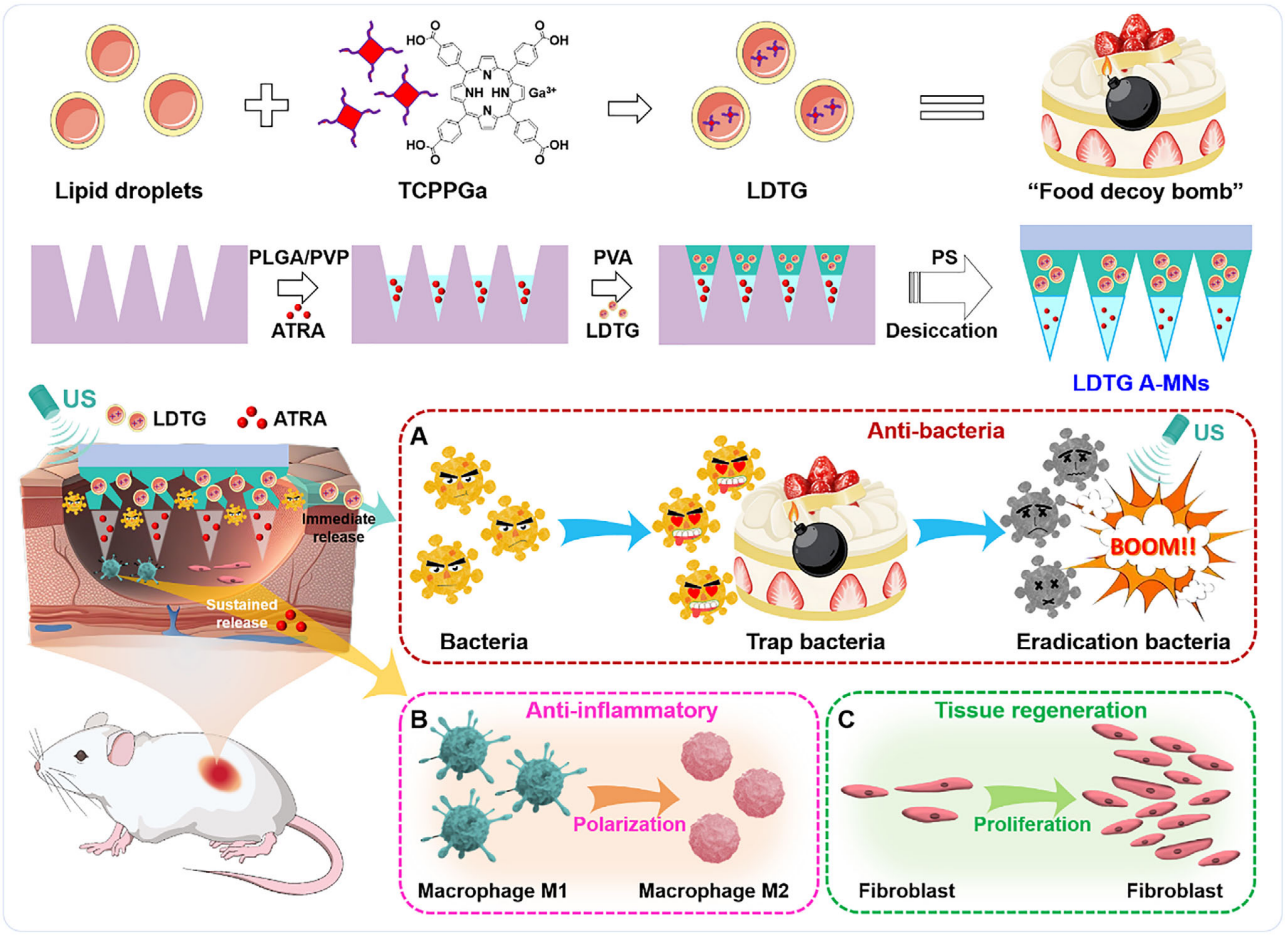
Schematic illustration of the fabrication and structural design of the LDTG A‐MN patch, and its therapeutic paradigm involving bacteria targeting and elimination, immune regulation, tissue regeneration, and enhanced wound healing.

Here, we developed an efficient and biocompatible dual‐functional MN patch, termed LDTG A‐MNs, to achieve synergistic antibacterial therapy and immunoregulatory wound repair (Scheme 1). The bilayer MN architecture enabled spatiotemporal separation of therapeutic functions, allowing rapid antibacterial action via LDTG release and sustained immunomodulation through controlled all‐trans retinoic acid (ATRA) delivery. The MN base layer—comprising rapidly dissolving polyvinyl alcohol (PVA) and sucrose—encapsulated a LD‐engineered gallium‐based sonodynamic platform (LDTG). Incorporation of LDs into the MN platform provided biomimetic bacterial targeting, enhancing pathogen capture and local retention of ROS‐generating LDTG, which surpassed the passive diffusion and limited targeting of conventional MN systems [24]. Upon skin insertion, the base layer rapidly dissolved in wound exudate, releasing LDTG to initiate an immediate “capture‐kill” antibacterial mechanism, which was further amplified by low‐frequency US (1 MHz) to generate abundant ROS for SDT‐mediated pathogen eradication and biofilm disruption. Meanwhile, the MN tip layer—constructed from poly(lactic‐co‐glycolic acid) (PLGA) and polyvinylpyrrolidone (PVP)—provided sustained release of ATRA, an established immunomodulator that promotes macrophage M2 polarization and facilitates tissue regeneration. In bacteria‐infected wound models, US‐activated LDTG A‐MNs achieved significant bacterial clearance, reduced inflammation, promoted collagen deposition, enhanced angiogenesis, and accelerated wound closure. Collectively, LDTG A‐MNs integrated pathogen targeting, sonodynamic disinfection, inflammation regulation, and pro‐regenerative therapy into a single minimally invasive platform, offering a promising alternative to conventional antibiotic‐based wound treatments.

## 2 Results and Discussion

### 2.1 Characterizations of LDTG

**FIGURE 1:**
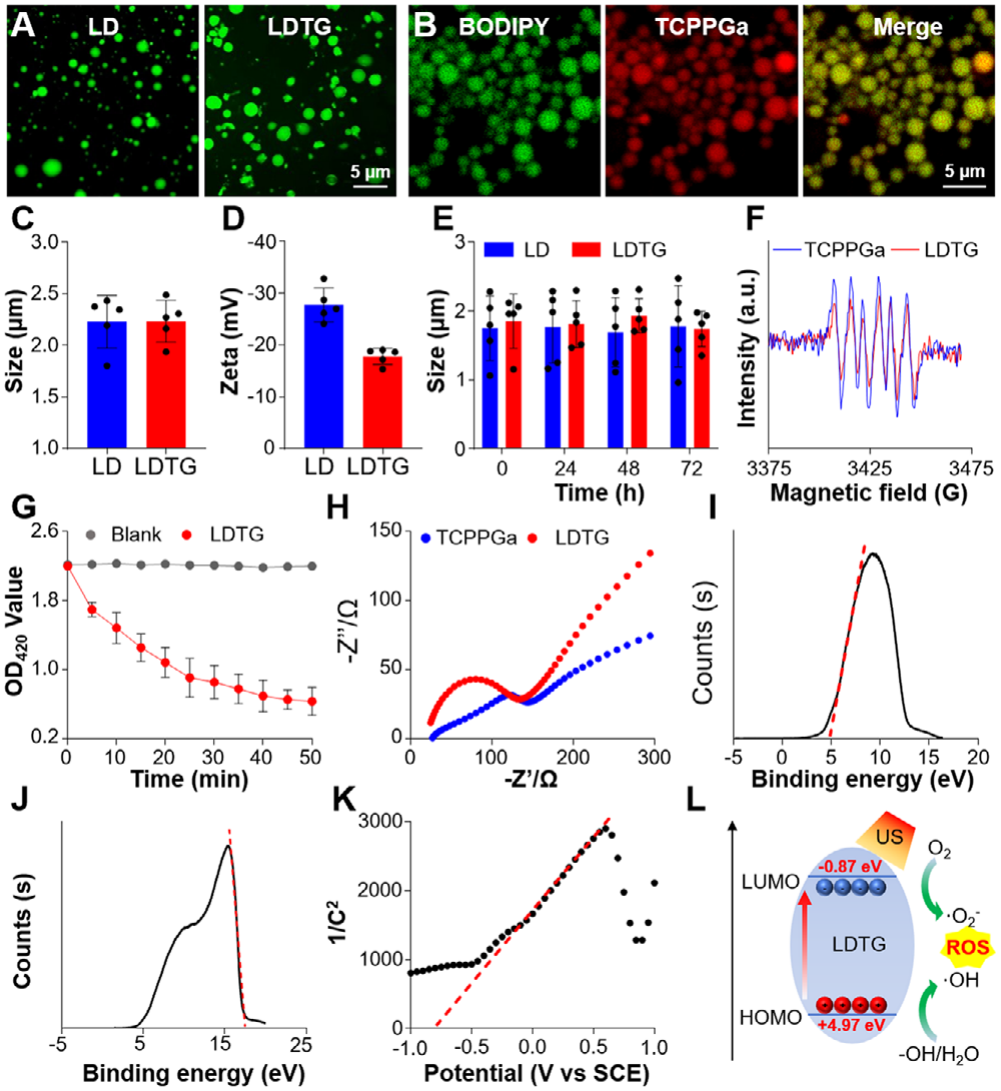
Characterization of LDTG. (A) Confocal fluorescence images of LD and LDTG. (B) Confocal images of LDTG under different excitation lights. (C) Hydrodynamic size distribution, and (D) zeta potential measurements of LD and LDTG. (E) Stability of LDTG determined by dynamic light scattering (DLS) over 72 h. (F) Electron spin resonance (ESR) spectra of •O2− of TCPPGa and LDTG after 10 min of US irradiation. (G) Total ROS generation of LDTG under USs irradiation detected using the DPBF probe. (H) Electrochemical impedance spectra of TCPPGa and LDTG. (I,J) Ultraviolet photoelectron spectroscopy (UPS) spectra, and (K) Mott–Schottky plots of LDTG. (L) US‐induced sonocatalytic mechanism of the LDTG heterostructure for enhanced ROS generation. Data are presented as mean ± SD (n = 5).

The sonosensitizer TCPPGa was first synthesized via a water‐based method [41]. Its successful preparation was confirmed by scanning electron microscopy (SEM) and X‐ray diffraction (XRD) (Figure S1). The synthesized TCPPGa showed good dispersibility in HEPES buffer, as indicated by its dispersion behavior and UV–vis spectra (Figure S2). Subsequently, TCPPGa was encapsulated into LDs through US‐assisted emulsification to form engineered LDTG. Confocal fluorescence imaging using the lipid‐specific dye BODIPY verified the presence of LDs before and after TCPPGa loading (Figure 1A,B). Optical microscopy and transmission electron microscopy (TEM) further demonstrated that the structural integrity of LD was preserved after encapsulation (Figure S3). Dynamic light scattering (DLS) measurements showed that LDTG exhibited an average hydrodynamic diameter of 2.23 ± 0.20 µm with a narrow size distribution (Figure 1C). Moreover, their zeta‐potential was measured to be −17.82 ± 1.55 mV, which is less negative than that of pristine LD (−27.76 ± 3.28 mV), likely due to the incorporation of positively charged regions of TCPPGa (Figure 1D). LDTG maintained colloidal stability in phosphate‐buffered saline (PBS) for at least 3 days without significant variations in particle size (Figure 1E).

To determine whether the encapsulated TCPPGa retained its sonodynamic activity, ROS production was evaluated. Using 5,5‐dimethyl‐1‐pyrroline‐N‐oxide (DMPO) as a probe, electron spin resonance spectra showed comparable •O_2_^−^ signal intensities between LDTG and free TCPPGa (Figure 1F), demonstrating that the loading process did not impair ROS‐generating capability. Additionally, total ROS generation was assessed using 1,3‐diphenylisobenzofuran (DPBF). Under US irradiation (1 MHz, 0.97 W/cm^2^, 50% duty cycle), the absorbance at 420 nm decreased significantly from 2.20 ± 0.08 to 0.64 ± 0.16 over 50 min, confirming efficient ROS production by LDTG (Figure 1G). Electrochemical impedance spectroscopy (EIS) revealed a larger semicircle radius for LDTG compared with TCPPGa (Figure 1H), indicating increased charge‐transfer resistance due to the lipid matrix. Ultraviolet photoelectron spectroscopy (UPS) further showed that the valence band maximum (VBM) and work function of LDTG were +3.97 and +6.42 eV, respectively (Figure 1I,J). Mott‐Schottky analysis demonstrated a positive slope, confirming that LDTG behaves as an n‐type semiconductor (Figure 1K), and the conduction band (CB) was estimated to be −0.87 eV. Based on these measurements, a band diagram was constructed to illustrate the US‐triggered ROS generation mechanism (Figure 1L). Upon US irradiation, LDTG generated electron‐hole pairs, thereby exhibiting a pronounced capacity for ROS production. It facilitated efficient charge transfer by capturing US‐excited electrons, promoting effective separation of electron‐hole pairs and preventing their recombination—an essential factor for enhancing charge reaction efficiency. The US‐activated electrons were transferred to surrounding O_2_ at the LDTG surface, producing •O_2_^−^, while the excited holes oxidized water to produce •OH, thereby initiating the SDT process for potential antibacterial applications. TCPPGa exhibited a certain bandgap, which was critical for its SDT activity (Figure S4). However, the encapsulation of TCPPGa in LDs (e.g., LDTG) slightly attenuated charge separation efficiency, likely due to the shielding effect of the LDs, which could influence the material's electronic properties and reduce charge transfer efficiency.

### 2.2 Bacteria‐Targeting Effect of LDTG In Vitro

**FIGURE 2:**
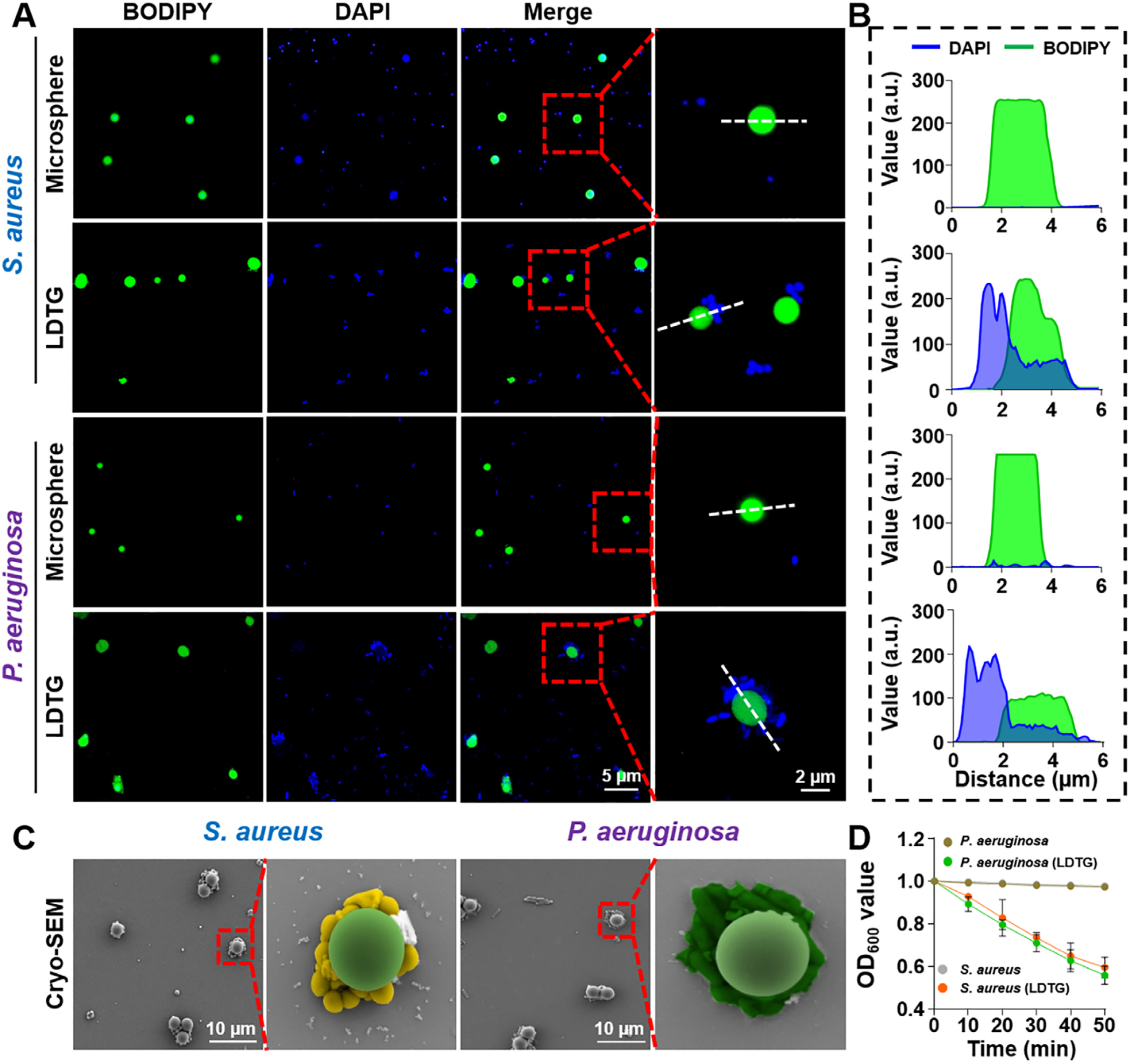
Bacterial capture performance of LDTG. (A) Fluorescence images of S. aureus and P. aeruginosa incubated with commercial microsphere or LDTG. (B) Quantitative fluorescence intensity analysis of bacterial regions (corresponding to dashed‐line ROIs in panel A) following incubation with microspheres or LDTG. (C) Cryo‐SEM images showing the morphological evidence of bacterial capture by LDTG. (D) Time‐dependent OD600 profiles of S. aureus and P. aeruginosa during incubation with LDTG, indicating progressive bacterial sequestration. Data are presented as mean ± SD (n = 3).

It has been reported that LDs serve as essential lipid sources for certain pathogens, which actively induce and associate with host LDs during infection [42]. Based on this biological affinity, we examined whether LDTG could selectively capture bacteria. To evaluate bacterial binding, *S. aureus* and *P. aeruginosa* were co‐incubated with LDTG and stained with DAPI (bacteria) and BODIPY (LDs), followed by fluorescence imaging. Commercial SiO_2_ microspheres with similar size and morphology were used as a non‐targeted control. Unlike LDs, which can be recognized and internalized by bacteria due to their role as nutrient reservoirs, SiO_2_ microspheres are inert inorganic particles and do not induce bacterial aggregation. As shown in Figure 2A, bacteria remained dispersed in the presence of commercial SiO_2_ microspheres, indicating a lack of intrinsic targeting ability. In contrast, distinct bacterial clustering was observed around LDTG, accompanied by strong co‐localized fluorescence signals (Figure 2B), confirming the enhanced pathogen‐binding capacity of LDTG. Cryo‐SEM imaging further validated these findings by directly visualizing the physical adherence of bacteria to the surface of LDTG (Figure 2C), demonstrating their strong bacterial capture capability. To quantitatively assess the capture efficiency, the optical density (OD) of bacterial suspensions was measured following incubation with LDTG over time. As shown in Figure 2D, a time‐dependent decrease in OD_600_ was observed in the LDTG groups, whereas the control groups displayed little change, indicating progressive sequestration of bacteria by LDTG. To further validate this observation and exclude potential interference from lipid‐induced sedimentation and light scattering, bacterial capture efficiency was additionally quantified using colony‐forming unit (CFU) assays. As shown in Figure S5, consistent results were obtained, confirming the effective removal of bacteria from the supernatant by LDTG.

### 2.3 Preparation and Characterization of LDTG A‐MNs

**FIGURE 3:**
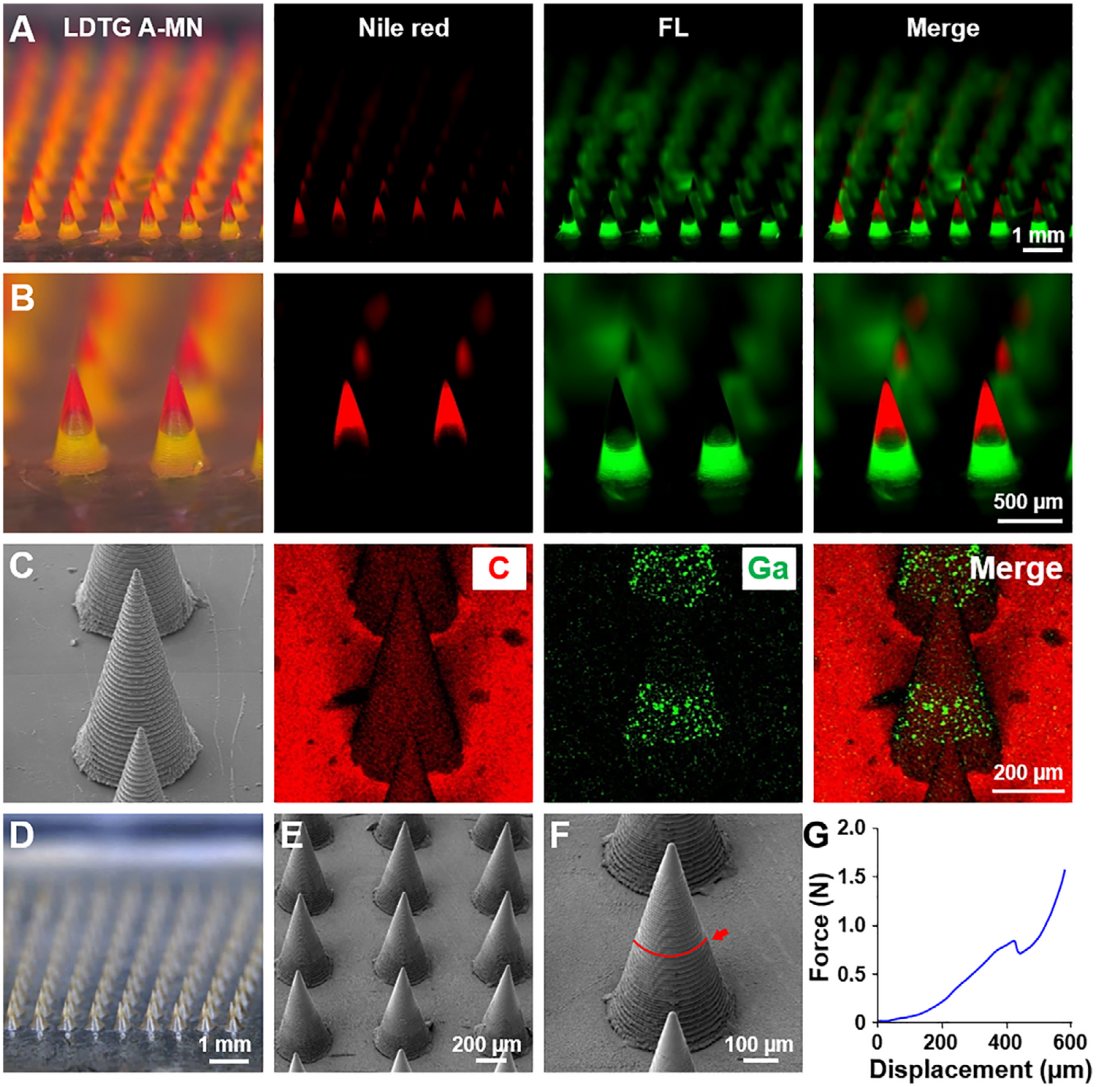
Characterization of multifunctional LDTG A‐MN patches. (A,B) Optical and magnified images of the bilayer and fluorescent images of LDTG A‐MNs. (C) Elemental mapping of LDTG A‐MNs. (D) Optical image and (E,F) SEM images of LDTG A‐MNs. The red dashed line marks the interface between the tip layer and the dissolvable base layer. (G) The force‐displacement curve of LDTG A‐MNs.

To enable minimally invasive and efficient delivery of both LDTG and ATRA into infected wound tissue, we fabricated a bilayer MN patch (termed LDTG A‐MNs) using a sequential casting approach. To validate the spatial distribution of each component within the MN architecture, Nile Red and fluorescein sodium (FL) were incorporated as fluorescent markers into the tip and base layers, respectively. Confocal fluorescence imaging confirmed the well‐defined bilayer structure, with the fluorescent signals clearly separated between the two layers (Figure 3A,B). Elemental mapping via energy dispersive spectroscopy (EDS) further verified that Ga signals originating from LDTG were confined to the base layer, while carbon‐rich components were uniformly distributed in the tip region (Figure 3C), confirming successful layer‐specific loading. The resulting LDTG A‐MN patches consisted of a 10 × 10 MN array covering an area of approximately 0.5 cm^2^ (Figure 3D). Each MN exhibited a conical morphology with an average height of ∼850 µm and a base diameter of ∼400 µm, displaying a distinct and continuous bilayer configuration (Figure 3E,F). Mechanical compression testing demonstrated that each MN sustained a fracture force of approximately 0.82 N per needle (Figure 3G), which exceeds the threshold generally required for reliable transdermal penetration [43]. These results collectively confirm that the LDTG A‐MNs possess adequate structural integrity and mechanical robustness for effective skin insertion and localized therapeutic delivery.

### 2.4 Drug Release From LDTG A‐MNs In Vitro

**FIGURE 4:**
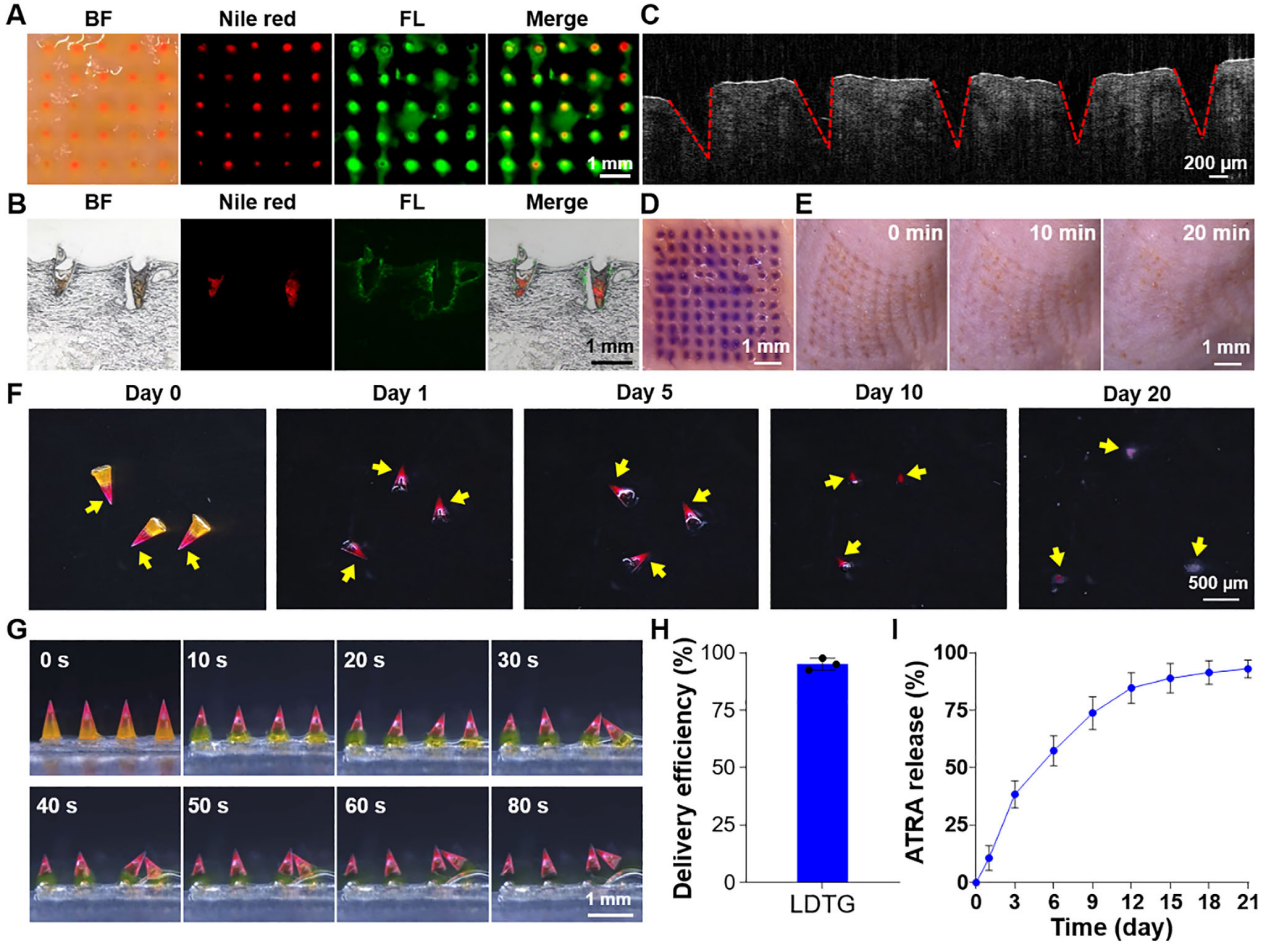
Evaluation of transdermal delivery and drug release performance of LDTG A‐MNs. (A) Representative bright‐field microscopy image of porcine skin after insertion of the LDTG A‐MN patch. (B) Histological sections confirming the successful embedding of LDTG A‐MNs into porcine skin ex vivo. (C) OCT images showing microchannels created in porcine skin after MN insertion. (D) Crystal violet‐stained porcine skin displaying puncture sites produced by the MNs. (E) Images of mouse skin recovery within 20 min post‐application of LDTG A‐MNs in vivo. (F) Inverted optical micrographs showing gradual degradation of MN tips over 20 days under simulated wound conditions. (G) Optical images demonstrating rapid dissolution of the water‐soluble MN base layer and separation of the tips within 80 s in PBS. (H) Delivery efficiency of LDTG following transdermal insertion using LDTG A‐MNs. (I) Cumulative in vitro release profile of ATRA from LDTG A‐MNs over 21 days. Data are presented as mean ± SD (n = 3).

Efficient skin penetration and controlled drug release are essential for the therapeutic performance of MN‐based delivery systems. To evaluate the transdermal insertion capability of the LDTG A‐MNs, the MN patches were applied to ex vivo porcine skin. Fluorescence imaging demonstrated that both fluorescent probes used to mimic LDTG and ATRA were successfully delivered into the skin (Figure 4A), which was further confirmed by histological cross‐sections showing dye distribution within the epidermis and dermis (Figure 4B). Moreover, optical coherence tomography (OCT) imaging revealed clear microchannel formation, verifying effective MN penetration (Figure 4C). Consistently, crystal violet staining of puncture sites in mouse skin confirmed successful and uniform MN insertion in vivo (Figure 4D). Notably, the microchannels gradually closed and were nearly invisible within 20 min post‐application, indicating rapid self‐sealing of the skin barrier (Figure 4E). The release behavior of LDTG and ATRA from the bilayer MN structure was subsequently examined. Fluorescence microscopy showed that the MN base layer dissolved within 80 s after contact with aqueous conditions (Figure 4G), enabling rapid release of LDTG at the wound interface. In contrast, the MN tips exhibited gradual degradation over approximately 20 days in a simulated wound environment (Figure 4F), providing a sustained release profile for ATRA. Quantitative analysis demonstrated delivery efficiency of 95.19 ± 2.55% for LDTG after skin insertion (Figure 4H). The cumulative release of ATRA reached 93.14 ± 3.86% by day 20 (Figure 4I), consistent with the prolonged degradation rate of the tip layer. Together, these results confirm that the LDTG A‐MNs enable rapid deposition of LDTG and long‐term controlled release of ATRA, a combination ideal for simultaneous antibacterial therapy and promotion of tissue regeneration.

### 2.5 Antibacterial and Anti‐Biofilm Activities of LDTG A‐MNs In Vitro

**FIGURE 5:**
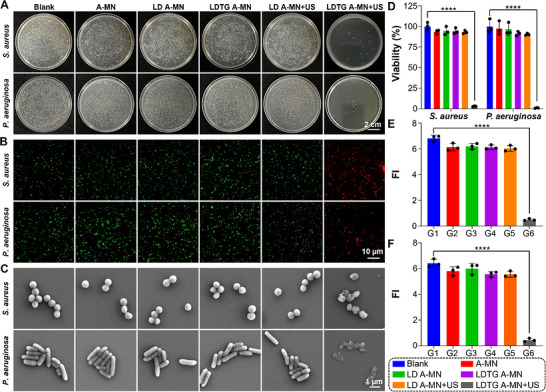
In vitro antibacterial performance of LDTG A‐MN patches. (A) Representative agar plate photographs showing the antibacterial activity of LDTG A‐MNs against S. aureus and P. aeruginosa. (B) Live/dead staining of S. aureus and P. aeruginosa after different treatments. (C) SEM images illustrating morphological changes in S. aureus and P. aeruginosa following the indicated treatments. (D) Quantitative bacterial viability after treatment with LDTG A‐MNs. (E,F) Mean fluorescence intensity (FI) of S. aureus and P. aeruginosa under different treatments. Data are presented as mean ± SD (n  =  3). ****p < 0.0001.

Given the efficient US‐activated ROS generation and bacteria‐binding capability of LDTG, the antibacterial performance of LDTG A‐MNs was systematically evaluated. As shown in Figure 5A,D, LDTG A‐MNs exhibited potent bactericidal activity against *S. aureus* and *P. aeruginosa*, achieving viability reductions of 96.81 ± 0.42% and 98.84 ± 0.38%, respectively, following US irradiation (1 MHz, 0.97 W/cm^2^, 50% duty cycle, 10 min). Live/dead staining using SYTO 9/PI further corroborated these findings, where the LDTG A‐MNs + US group displayed predominantly red fluorescence, indicating extensive bacterial cell death (Figure 5B,E,F). SEM imaging revealed marked morphological disruption of bacterial cell membranes after treatment, in contrast to the intact and smooth surfaces observed in untreated controls (Figure 5C), confirming that LDTG A‐MNs effectively induce membrane damage through US‐triggered ROS generation.

**FIGURE 6:**
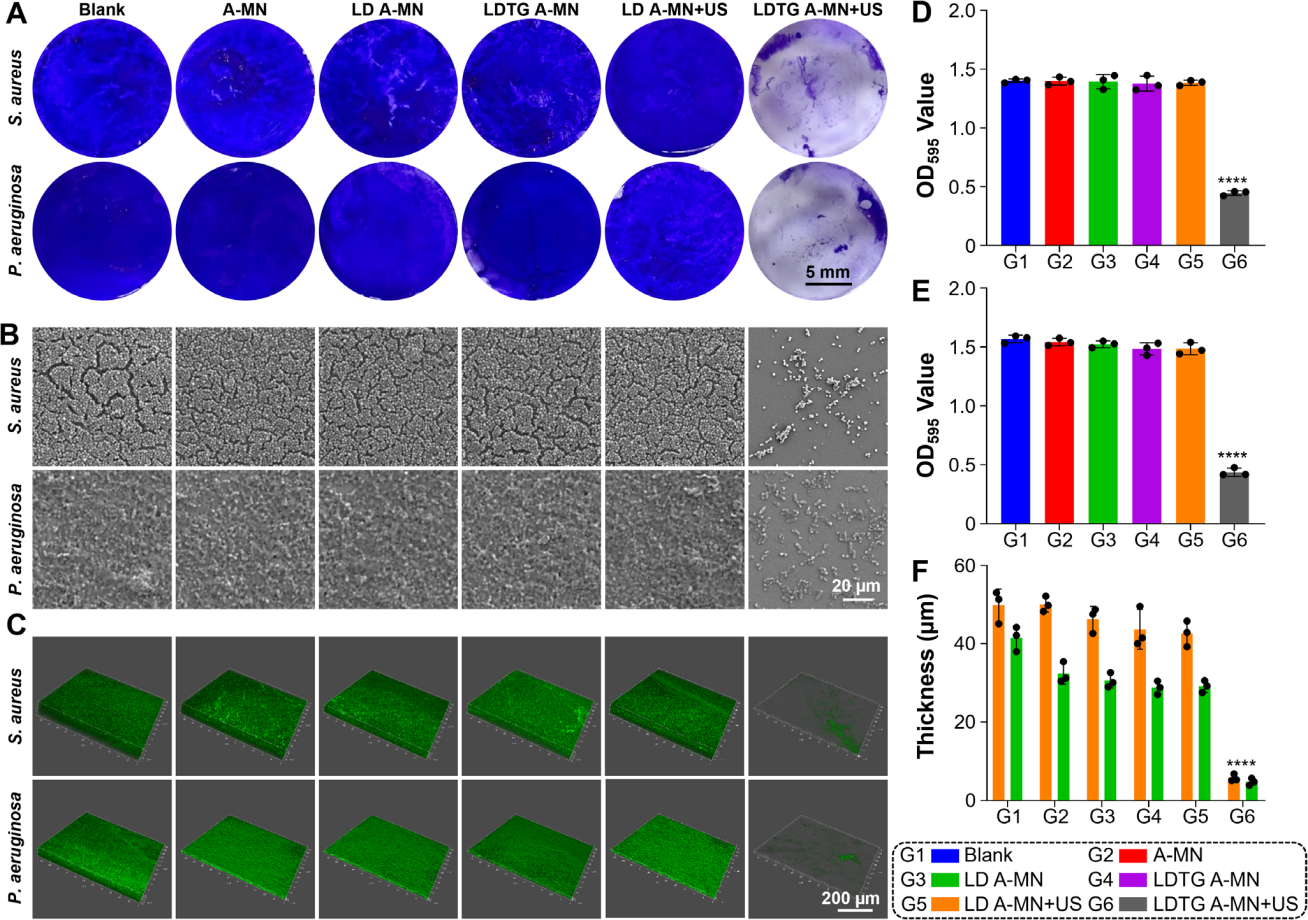
Anti‐biofilm efficacy of LDTG A‐MNs against S. aureus and P. aeruginosa. (A) Representative images of immature biofilms stained with crystal violet following different treatments. (B) SEM images showing morphological changes in immature biofilms after treatment. (C) Confocal fluorescence images and corresponding 3D reconstructions of acridine orange (AO)‐stained immature biofilms after various treatments. (D,E) Quantification of biofilm biomass by OD_595_ measurements for S. aureus and P. aeruginosa mature biofilms. (F) Biofilm thickness of each group determined by confocal Z‐stack analysis. Data are presented as mean ± SD (n  =  3). ****p < 0.0001.

In chronic infected wounds, dense bacterial biofilms significantly hinder antimicrobial penetration and therapeutic efficacy. Therefore, we further assessed whether LDTG A‐MNs could disrupt mature biofilms. Crystal violet staining demonstrated that US‐activated LDTG A‐MNs markedly reduced biofilm biomass for both *S. aureus* and *P. aeruginosa* (Figure 6A), which was quantitatively confirmed by decreased OD_595_ values (Figure 6D,E). SEM analysis showed that untreated biofilms exhibited dense, multilayered extracellular matrices with compact architecture, whereas LDTG A‐MNs + US treatment resulted in extensive film detachment, fragmentation, and collapse of structural integrity (Figure 6B). Additionally, 3D confocal laser scanning microscopy using acridine orange staining revealed that biofilms were nearly completely eliminated following combined treatment (Figure 6C,F). Although MN penetration might contribute to partial mechanical disruption of superficial biofilm layers, the limited antibacterial and anti‐biofilm effects observed in MN‐only groups indicated that mechanical action alone was insufficient. The pronounced biofilm eradication was primarily attributed to US‐triggered ROS generation, with MNs facilitating efficient delivery and penetration. Additionally, the release of Ga ions from LDTG over 72 h was measured to be only 12.23 ± 0.89 µM (Figure S6), a concentration insufficient to induce significant ferroptosis or iron‐mimicking antibacterial effects. Therefore, while trace Ga might contribute minimally, the potent bactericidal activity of LDTG A‐MNs was predominantly mediated by US‐triggered ROS generation.

Taken together, these results demonstrate that LDTG A‐MNs not only kill planktonic bacteria through US‐activated ROS production but also effectively disrupt established biofilms, highlighting their strong potential for resolving biofilm‐associated wound infections.

### 2.6 Biosafety and Anti‐Inflammatory Effects of LDTG A‐MNs

**FIGURE 7:**
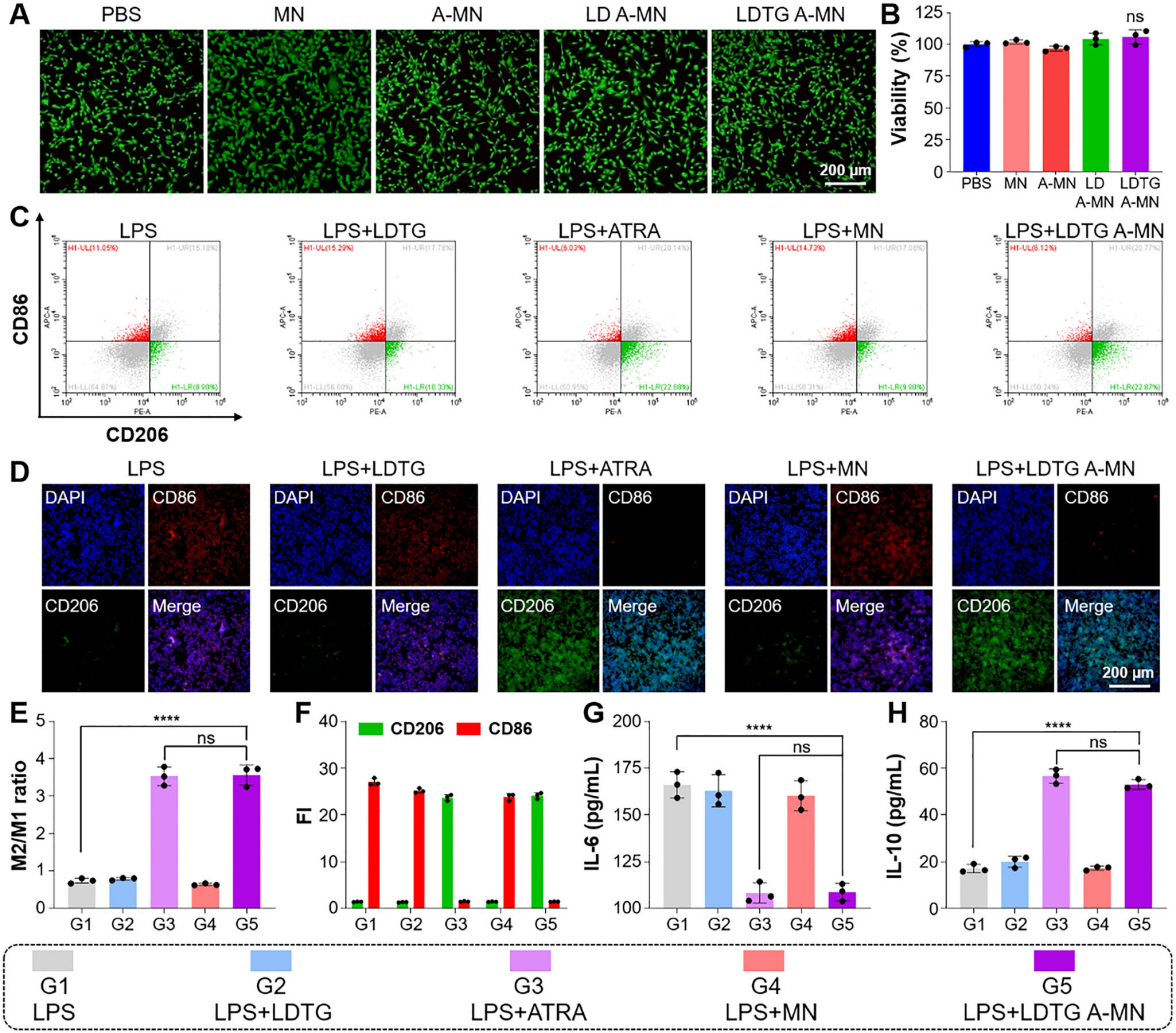
Cytocompatibility and macrophage polarization induced by LDTG A‐MNs. (A) Live/dead staining of NIH‐3T3 cells after treatment with different samples. (B) Cell viability of NIH‐3T3 cells evaluated by MTT assay. (C) Representative flow cytometry plots showing macrophage polarization (CD86 for M1, CD206 for M2). (D) Representative immunofluorescence images of RAW264.7 macrophages stained for CD86 (M1) and CD206 (M2). (E) Quantification of the M2/M1 macrophage ratio. (F) Quantification of fluorescence intensity (FI) for CD86 and CD206. (G,H) ELISA analysis of IL‐6 (G) and IL‐10 (H) secretion in LPS‐induced RAW264.7 cells following different treatments. Data are presented as mean ± SD (n  = 3). ****p < 0.0001. The ns indicates no significance.

The biocompatibility of LDTG A‐MNs was first evaluated in vitro. Live/dead staining using Calcein‐AM (green) and Propidium Iodide (PI) (red) revealed that NIH‐3T3 cells treated with LDTG A‐MNs exhibited predominantly green fluorescence, comparable to the PBS‐treated control group (Figure 7A). Consistently, MTT assays showed that cells cultured with LDTG or LDTG A‐MNs for 24 h maintained high viability (Figure 7B; Figure S7), confirming minimal cytotoxicity and excellent cellular compatibility. The biocompatibility was further validated by hemolysis test (Figure S8). The ability of LDTG A‐MNs to support cell migration and proliferation was examined using a scratch assay. NIH‐3T3 cells cultured with LDTG A‐MN extract exhibited accelerated migration into the wound gap over time (Figure S9), demonstrating favorable cell compatibility and wound repair‐promoting capacity. Macrophage polarization plays a central role in modulating inflammation during wound healing. Flow cytometry analysis revealed that LDTG A‐MNs treatment significantly increased CD206^+^ M2 macrophage populations while reducing CD86^+^ M1 populations compared with the LPS‐stimulated group (Figure 7C,E). Immunofluorescence staining further supported these results, where the LDTG A‐MNs group exhibited enhanced green fluorescence corresponding to CD206 and diminished red fluorescence corresponding to CD86 (Figure 7D,F). In addition, ELISA results showed that LDTG A‐MNs treatment suppressed the production of the pro‐inflammatory cytokine IL‐6 while upregulating the anti‐inflammatory cytokine IL‐10 (Figure 7G,H). Collectively, these findings demonstrate that LDTG A‐MNs possess excellent biosafety and effectively promote macrophage polarization toward an anti‐inflammatory M2 phenotype, thereby supporting a wound microenvironment conducive to tissue regeneration.

### 2.7 Therapeutic Efficacy of LDTG A‐MNs for Infected Wound Management In Vivo

**FIGURE 8:**
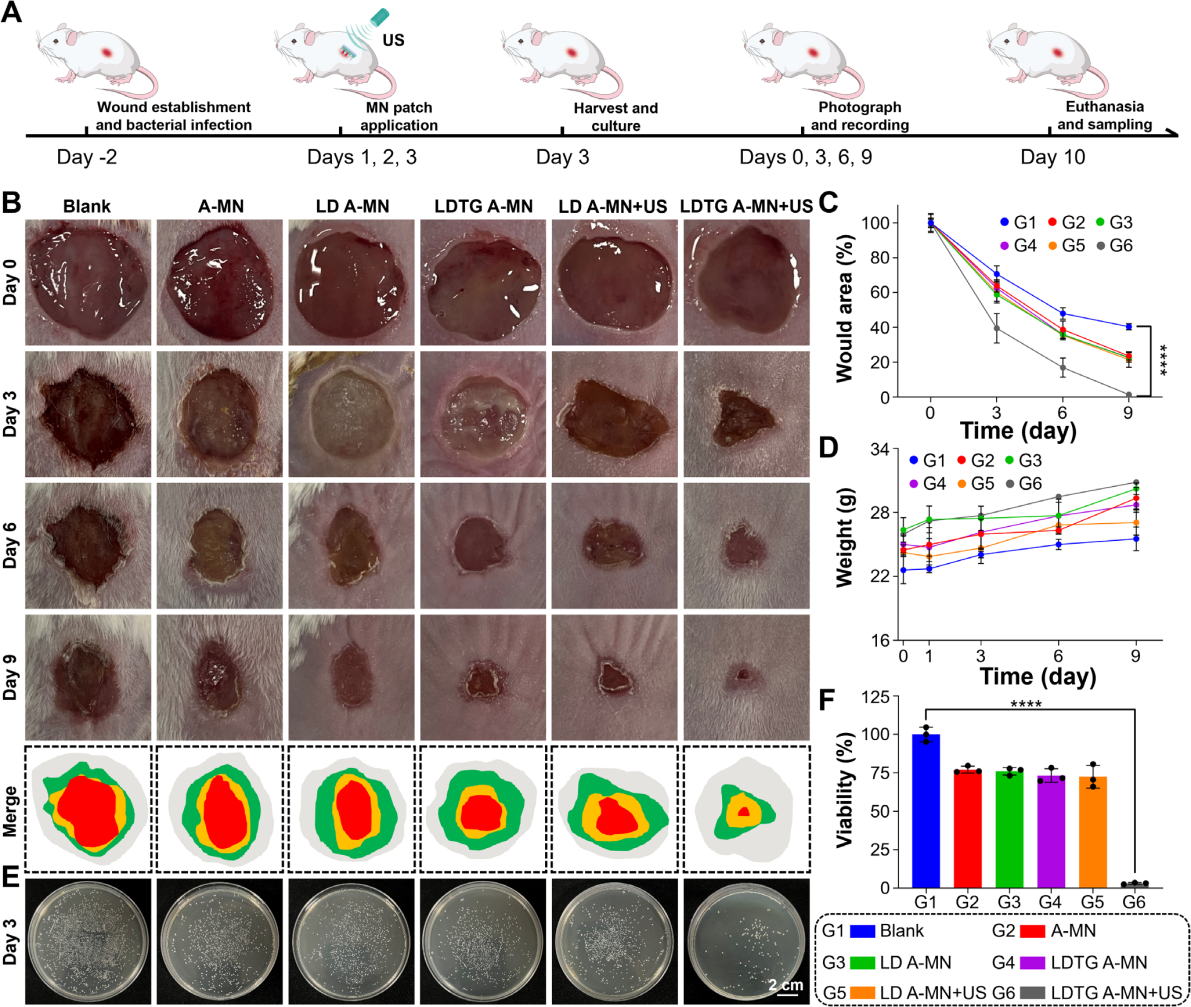
In vivo antimicrobial efficacy and wound‐healing performance of LDTG A‐MNs. (A) Schematic illustration of the S. aureus‐infected murine wound model and the treatment protocol. (B) Representative photographs of wound appearance on days 0, 3, 6, and 9 under different treatments. (C) Quantitative analysis of wound closure over 9 days (n = 5). (D) Body‐weight changes of mice during treatment, indicating systemic biosafety (n = 5). (E) Representative photographs of bacterial colonies cultured from wound tissue collected on day 3. (F) Quantification of bacterial viability in wound beds across different groups (n = 3). Data are presented as mean ± SD. ****p < 0.0001.

The therapeutic potential of LDTG A‐MNs in promoting infected wound healing was evaluated in a *S. aureus*‐infected murine skin defect model (Figure 8A). Although in vitro experiments confirmed that LDTG A‐MNs effectively eradicated both Gram‐positive (*S. aureus*) and Gram‐negative (*P. aeruginosa*) bacteria, the in vivo studies focused on *S. aureus* to align with ethical considerations and minimize animal use. Mice were randomly divided into six groups (*n* = 5 per group), and US treatment (1 MHz, 0.97 W/cm^2^, 50% duty cycle, 10 min/day) was administered for three consecutive days where indicated. Wound healing was monitored for 9 days, and representative images were recorded on days 0, 3, 6, and 9 (Figure 8B). By day 3, the LDTG A‐MNs + US group displayed markedly reduced purulent exudate and attenuated local inflammation compared with all non‐US treatment groups, attributable to US‐activated ROS‐mediated bacterial clearance. To further quantify antibacterial efficacy, wound tissues were collected after three days of treatment, homogenized, and plated for bacterial colony analysis (Figure 8E). The LDTG A‐MNs + US group exhibited the lowest colony‐forming units (CFUs), indicating significantly enhanced bacterial eradication (Figure 8F). Over the full 9‐day treatment period, mice in the LDTG A‐MNs + US group demonstrated the fastest wound closure and most complete tissue repair (Figure 8C), highlighting the synergistic contributions of US‐triggered bactericidal activity and sustained ATRA release to support tissue regeneration. Additionally, no significant differences in body weight were observed among the groups throughout the study (Figure 8D), confirming good systemic biosafety of the MNs treatment. Temperature monitoring during US irradiation revealed only a slight increase under the applied conditions (Figure S10), indicating that thermal effects were negligible and that the observed therapeutic outcomes were primarily attributed to sonodynamic ROS generation. Although LDTG exhibited a micron‐scale size, its delivery relied on MN‐mediated localized deposition rather than passive diffusion. Following MN insertion, LDTG rapidly dispersed within the wound microenvironment, achieving effective spatial distribution as confirmed by fluorescence imaging (Figure S11). The polymer matrix (PVA, PLGA, and PVP) primarily served as a biocompatible delivery scaffold. Although it might provide a moist microenvironment that was beneficial for wound healing, control experiments indicated that the observed therapeutic effects were predominantly attributed to LDTG‐mediated antibacterial activity and ATRA‐driven immunoregulation. Overall, these findings demonstrate that LDTG A‐MNs, when combined with US activation, provide effective antibacterial therapy while simultaneously promoting wound regeneration, offering a promising strategy for treating infected and biofilm‐associated wounds.

### 2.8 Evaluation of Tissue Regeneration, Inflammatory Response, and Angiogenesis After Treatment With LDTG A‐MNs In Vivo

**FIGURE 9:**
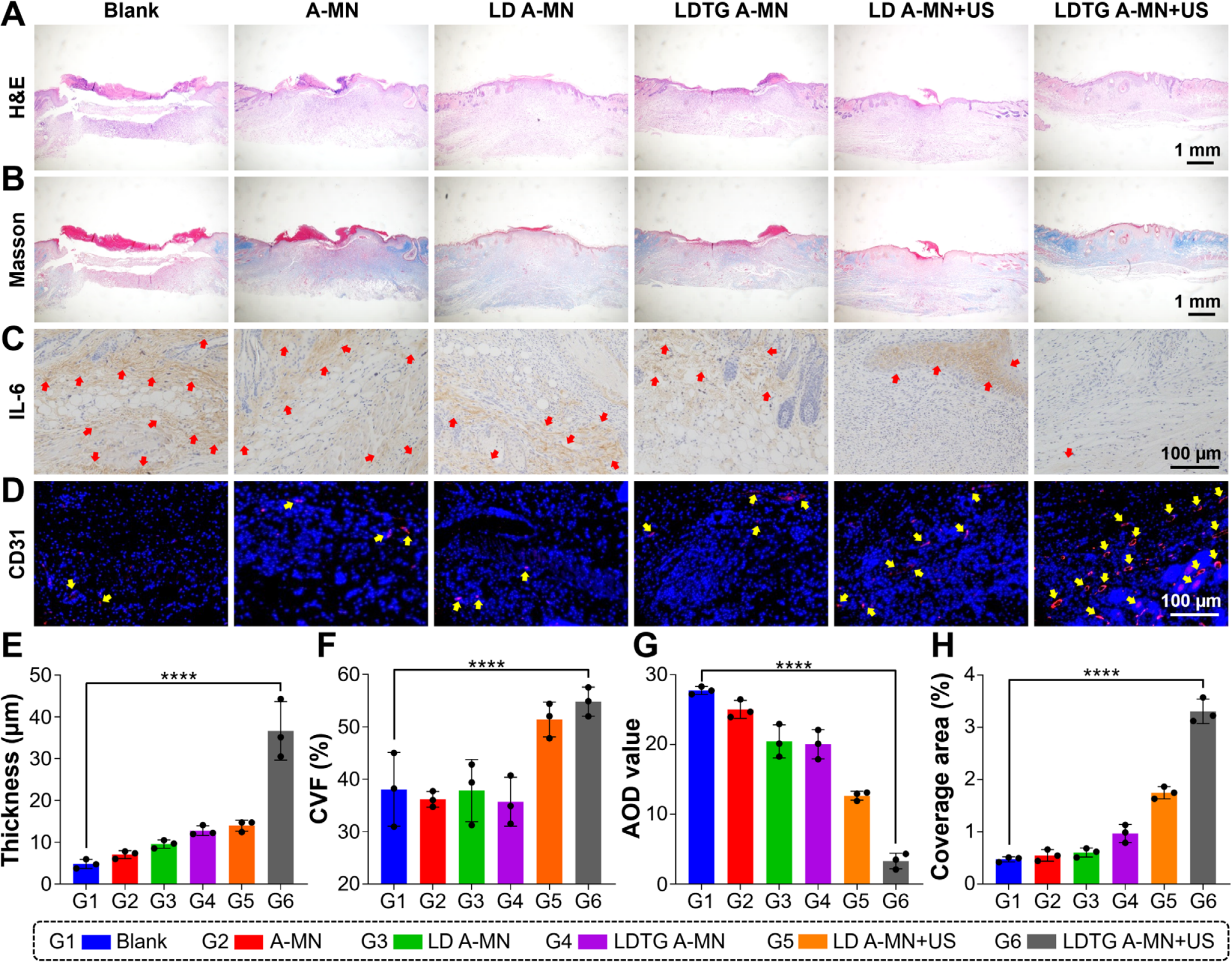
Assessment of tissue regeneration, inflammatory response, and angiogenesis post‐treatment. (A) H&E staining for general tissue morphology and epithelial thickness. (B) Masson's trichrome staining for collagen deposition. (C) IL‐6 immunohistochemical staining for inflammation (red arrows indicate IL‐6 positive cells). (D) Immunofluorescence images of CD31 staining for neovascularization (yellow arrows indicate blood vessels; nuclei stained with DAPI, blue). (E) Quantitative analysis of regenerated epidermal thickness across treatment groups. (F) Quantification of collagen volume fraction (CVF) from Masson's staining. (G) Quantification of IL‐6 expression based on average optical density (AOD) from immunohistochemistry. (H) Quantification of CD31‐positive area, representing vascular density. Data are presented as mean ± SD (n  =  3). ****p < 0.0001.

To further assess the therapeutic benefits of LDTG A‐MNs, we examined tissue regeneration and inflammatory modulation at the wound sites. Hematoxylin and eosin (H&E) staining revealed varying degrees of re‐epithelialization among the treatment groups (Figure 9A). Notably, the LDTG A‐MN + US group exhibited the most complete epithelial regeneration, with an average epithelial thickness of 36.65 ± 7.00 µm, significantly greater than that in the other groups (Figure 9E), suggesting accelerated wound closure and tissue reconstruction. Masson's trichrome staining demonstrated enhanced collagen deposition in the regenerated tissue following treatment with LDTG A‐MNs under US (Figure 9B). Quantitative analysis confirmed that the collagen volume fraction (CVF) in the LDTG A‐MN + US group reached 54.81 ± 2.77% (Figure 9F), substantially higher than in all control groups. These results indicate improved extracellular matrix remodeling and maturation of newly formed tissue. To evaluate the inflammatory status of the wound microenvironment, IL‐6 expression was quantified using immunohistochemical staining. The LDTG A‐MN + US group displayed significantly reduced IL‐6 levels, with an average optical density (AOD) of 3.30 ± 1.12 (Figure 9C,G), indicating effective suppression of excessive inflammation induced by bacterial infection. Moreover, LDTG A‐MN + US treatment markedly increased IL‐10 expression and promoted M2 macrophage polarization, as shown by elevated CD206 and reduced CD86 levels in wound tissues (Figure S12). These results confirmed that LDTG A‐MNs effectively modulated the wound immune microenvironment to support anti‐inflammatory responses and healing. Importantly, although ATRA had been reported to have context‐dependent pro‐inflammatory effects, its sustained and localized release from the MN tips, combined with prior bacterial clearance by LDTG‐mediated SDT, likely promoted resolution of inflammation and supported tissue regeneration rather than exacerbating the inflammatory response. Neovascularization, a key feature of functional wound repair, was examined via immunofluorescence staining for CD31. The LDTG A‐MN + US group exhibited markedly increased microvessel density (Figure 9D,H), highlighting enhanced angiogenesis and active tissue regeneration.

Furthermore, H&E staining of major organs (heart, liver, spleen, lung, and kidney) demonstrated no observable pathological abnormalities (Figure S13), confirming that the treatment exhibited no apparent systemic toxicity. Collectively, these results demonstrate that LDTG A‐MNs, when combined with US activation, promote robust epithelial regeneration, enhance collagen remodeling, reduce inflammatory responses, and stimulate angiogenesis, ultimately leading to improved healing outcomes in bacteria‐infected wounds.

## 3 Conclusion

In this work, we developed a lipid‐droplet‐engineered sonodynamic MN platform (LDTG A‐MNs) that enables synergistic bacterial eradication and accelerated wound regeneration. By leveraging the natural bacterial tropism of LD and the US‐responsive ROS generation of TCPPGa, the engineered LDTG demonstrated efficient bacterial capture and potent sonodynamic bactericidal activity. Integration of LDTG with ATRA into a bilayer MN system enabled rapid release of LDTG at the wound site and sustained ATRA delivery to promote tissue remodeling. The bilayer design facilitated a temporally coordinated therapeutic sequence, where rapid antibacterial activity preceded sustained ATRA‐mediated immunomodulation, thereby minimizing the risk of premature M2 polarization. The resulting LDTG A‐MNs exhibited excellent mechanical robustness, biosafety, and enhanced cell proliferation and M2 macrophage polarization in vitro. In bacteria‐infected wound models, US‐activated LDTG A‐MNs achieved significant bacterial clearance, reduced inflammation, promoted collagen deposition, enhanced angiogenesis, and accelerated wound closure. Collectively, this work establishes a multifunctional, minimally invasive, and transdermally deliverable therapeutic platform that integrates pathogen targeting, sonodynamic antibacterial therapy, and immune‐regulated tissue regeneration, offering a promising strategy for the treatment of chronic, drug‐resistant, and complex infected wounds.

## 4 Experimental Section

### 4.1 Materials

The reagents sucrose, KCl, NaCl, Na_2_HPO_4_, KH_2_PO_4_, HCl, tricine, HEPES, KOH, TCPP, and Ga(NO_3_)_3_·H_2_O were purchased from Aladdin Reagent Co. (Shanghai, China). Fluorescein 5‐isothiocyanate, rhodamine B (RhB), MgCl_2_, dimethyl sulfoxide (DMSO), PVA, PVP, PLGA, and phenylmethanesulfonyl fluoride (PMSF) were obtained from Macklin Biochemical Technology Co. (Shanghai, China). Methanol, gelatin, agarose and glucose were acquired from Sinopharm Chemical Reagent Co. (Shanghai, China). LB nutrient broth and LB nutrient agar were sourced from Hopebio Biotechnology Co. (Qingdao, China). The Live/Dead BacLight bacterial viability kit was purchased from Thermo Fisher Scientific (USA). Dulbecco's Modified Eagle Medium (DMEM), fetal bovine serum, and penicillin‐streptomycin antibiotics were obtained from Procell Life Science & Technology Co. (Wuhan, China). The MTT cell proliferation and cytotoxicity assay kit, Calcein/PI cell viability/cytotoxicity assay kit were purchased from Beyotime Biotechnology Co. (Shanghai, China). The IL‐10 ELISA kit was acquired from Biotechnology Co. (Wuhan, China), and the IL‐6 ELISA kit was purchased from Bioswamp Biotechnology Co. (Wuhan, China). Deionized water was generated using a Milli‐Q Plus 185 water purification system (Millipore).

### 4.2 Preparation of TCPPGa

The optimized synthesis of TCPPGa was conducted in a 2 mL batch reactor by combining TCPP (15 mg), DMF (724 µL), and Ga(NO_3_)_3_·H_2_O (37.8 µL of a 1 M aqueous solution). The mixture was heated at 120°C for 48 h and subsequently cooled to room temperature over 6 h. The resulting solid was collected and washed twice with DMF and acetone to afford TCPPGa in 81% yield (204.8 mg, based on TCPP).

### 4.3 Preparation and Characterization of LDTG

A lipid droplet extraction solution was prepared using Buffer A (20 mM Tricine, 250 mM sucrose) and Buffer B (20 mM HEPES, 100 mM KCl, 2 mM MgCl_2_, pH 7.4), supplemented with a DMSO solution containing 0.25 M PMSF. Mice were euthanized, and their livers were immediately excised and minced in the PMSF solution. The tissue was homogenized on ice using a 200‐mesh sieve and then transferred into a centrifuge tube containing Buffer A. The homogenate was centrifuged at 2000 × g for 5 min, resulting in a white lipid droplet layer forming at the surface. The lipid droplets were collected, resuspended in Buffer B, and further purified by centrifugation, repeating this process three times to obtain high‐purity lipid droplets. Subsequently, TCPPGa was added to the purified lipid droplets, and the mixture was sonicated at 50 kHz for 6 h to yield the engineered lipid droplets (LDTG).

### 4.4 Measurement of ROS Generation by LDTG In Vitro

ROS generation was evaluated using 1,3‐diphenylisobenzofuran (DPBF) as a singlet oxygen probe [44]. Equal volumes of LDTG (100 µg/mL) and a DPBF‐containing DMSO solution were mixed and subjected to US irradiation (1 MHz, 0.97 W/cm^2^, 50% duty cycle) for 50 min. During irradiation, the absorbance of DPBF at 420 nm was measured at 10 min intervals to monitor ROS production.

### 4.5 Preparation of LDTG A‐MNs

LDTG‐loaded ATRA MNs (LDTG A‐MNs) were fabricated through a two‐step process involving high‐speed centrifugation and vacuum casting. First, ATRA (1 mg) was dissolved in 1 mL of a Dimethylacetamide (DMAC) solution containing 15% PLGA and 1.5% PVP to obtain the first‐layer solution. A 100 µL aliquot of this solution was added to a polydimethylsiloxane (PDMS) mold and centrifuged at 4200 rpm for 5 min to fill the MN tip cavities. For the second layer, LDTG was dispersed in an 18% PVA aqueous solution (1 mg/mL) to form a uniform suspension. Then, 100 µL of this suspension was added to the mold pre‐cast with ATRA, followed by the addition of 100 µL of a 20% polystyrene (PS) solution to form the backing layer. The mold was placed on a vacuum chuck at ‐0.1 MPa for 2 h and subsequently dried in a desiccator at room temperature for 72 h. After demolding, the LDTG A‐MN patch was obtained. Bright‐field images were captured using a fluorescence microscope (Olympus SZX16), and the MN microstructure was examined via field‐emission scanning electron microscopy (FE‐SEM, Tescan CLARA). The mechanical properties of the MNs were evaluated using a mechanical testing machine (Mark‐10 ESM303). To evaluate the release behavior of ATRA from LDTG A‐MNs, the MN patch was immersed in phosphate‐buffered saline (PBS, pH 5.6) at 37°C. At predetermined time intervals, aliquots of the supernatant were collected, and the released ATRA was quantified using a UV–vis spectrophotometer.

### 4.6 Delivery Efficiency of LDTG via MNs

The delivery efficiency of LDTG was calculated using the following method. Briefly, the MN patch was vertically inserted into ex vivo porcine skin, and thumb pressure was applied for 2 min. After removal of the patch, it was dissolved in DMSO to extract the TCPPGa from LDTG. The resulting solution was centrifuged at 3000 rpm for 10 min, and the supernatant was collected. The absorbance of the supernatant was measured at 420 nm using a UV–vis spectrophotometer, and the LDTG delivery efficiency was calculated based on a standard calibration curve. The delivery efficiency was determined using the following formula: Delivery efficiency (%) = (C1 × V1 – C2 × V2) × 100% / C1 × V1, where C1 is the LDTG concentration before insertion, V1 is the volume of the solution before insertion, C2 is the LDTG concentration after insertion, and V2 is the volume of the solution after insertion.

### 4.7 Bacterial Capture Ability of LDTG

The bacterial capture capability of LDTG against *S. aureus* and *P. aeruginosa* was evaluated as follows. Bacterial suspensions (10^8^ CFU/mL) were incubated with LDTG (100 µg/mL) for 1 h to allow bacterial adhesion. Subsequently, DAPI and BODIPY fluorescent dyes (10 µg/mL each) were added and incubated in the dark for 15 min. Inorganic SiO_2_ microspheres treated under identical conditions were used as the control. Fluorescence images were acquired and analyzed using a fluorescence microscope and ImageJ software. To further quantify bacterial capture, suspensions of *S. aureus* or *P. aeruginosa* with an initial optical density of OD_600_ = 0.6 were incubated with LDTG (1 mL) at 37°C for 1 h. After centrifugation at 1000 rpm for 5 min, the supernatant was collected and the OD_600_ values were recorded at predetermined time intervals using a microplate reader. Bacterial suspensions in PBS served as the control group.

### 4.8 In Vitro Antibacterial Performance of LDTG A‐MNs

First, the LDTG A‐MN patch was subjected to ultraviolet disinfection. The prepared MN extraction solution was mixed with 100 µL of a 10^7^ CFU/mL bacterial solution, and PBS was added to adjust the volume to 1 mL. After ultrasonic irradiation at 1 MHz, 0.97 W/cm^2^, 50% duty cycle for 10 min, 10 µL of the treated liquid medium was added to LB solid medium, and bacteria were evenly spread using a spreader. Finally, the solid medium was incubated at 37°C for 24 h, after which photographs were taken and bacterial colonies were counted. The relative bacterial viability was determined using the equation:

$$\mathrm{Bacterial}\mathrm{viability}\left(\%\right)=N_{t}/N_{c}\times 100\%$$

where *N*_t_ and *N*_c_ represented the number of bacterial colonies formed in the treatment group and the control group, respectively.

Subsequently, live/dead fluorescent staining was used as a reliable method to assess antibacterial properties. Bacteria in the logarithmic growth phase were collected and washed with PBS solution three times. The MN patch extraction solution was then added, followed by ultrasonic irradiation at 1 MHz, 0.97 W/cm^2^, 50% duty cycle for 10 min, and incubation at 37°C for 4 h. Afterward, a mixture of SYTO‐9 and PI was added and incubated in the dark for 30 min. Finally, the bacterial suspension was carefully applied to a glass slide, and fluorescence was examined using confocal laser scanning microscopy (CLSM, Nikon, A1). After incubation with the LDTG A‐MN patches, the bacterial morphology was also analyzed to evaluate the antibacterial properties of the MN patches using a field emission scanning electron microscope. (FE‐SEM, Carl Zeiss, Zeiss SIGMA).

### 4.9 Evaluation of Biofilm Disruption by LDTG A‐MNs

The ability of LDTG A‐MN patches to disrupt mature bacterial biofilms was assessed using *S. aureus* and *P. aeruginosa*. Bacterial suspensions (2 mL) were added to 6‐well plates and allowed to form biofilms. Extracted solutions of the MN patches were then applied, followed by ultrasound irradiation (1 MHz, 0.97 W/cm^2^, 50% duty cycle) for 10 min, and the plates were subsequently incubated at 37°C for 48 h. After incubation, the wells were gently washed three times with PBS, stained with 1 mL of crystal violet for 15 min, and rinsed to remove excess dye. The bound dye was dissolved in ethanol, and the absorbance at 595 nm was quantified using a microplate reader (TECAN M200PRO). For imaging, treated biofilms were stained using the AO kit and visualized by confocal laser scanning microscopy (CLSM). For morphological analysis, biofilms were fixed with 4% paraformaldehyde for 15 min and dehydrated through a graded ethanol series (60%, 70%, 80%, 90%, 100%; 15 min each). The samples were then sputter‐coated with gold and examined using field‐emission scanning electron microscopy (FE‐SEM, Tescan CLARA).

### 4.10 Evaluation of the Biocompatibility of LDTG A‐MNs

The biocompatibility of LDTG A‐MNs was assessed using NIH‐3T3 fibroblasts. Cytotoxicity of the MN components was evaluated via MTT assay and calcein‐AM/PI staining. Cell viability was calculated using the formula: Cell viability = (OD_treat_ – OD_blank_) / (OD_0_ – OD_blank_) ×100%, where OD_treat_ is the absorbance of the treated group, OD_blank_ is the absorbance of the blank (medium only), and OD_0_ is the absorbance of the untreated control group. To further examine hemocompatibility, rat blood was collected in anticoagulant tubes and centrifuged at 1500 rpm for 5 min at 4°C to isolate red blood cells (RBCs). The RBCs were washed and resuspended in PBS. A 200 µL aliquot of RBC suspension was mixed with PBS containing the MN patch. PBS and ultrapure water served as the negative and positive controls, respectively. The suspensions were incubated at room temperature for 4 h and then centrifuged at 3000 rpm for 10 min. The absorbance of the supernatant at 540 nm was measured using a UV–vis spectrophotometer, and the hemolysis rate was calculated.

### 4.11 Assessment of the Cell Migration‐Promoting Ability of LDTG A‐MNs

NIH‐3T3 cells were seeded in 6‐well plates and cultured in DMEM at 37°C with 5% CO_2_ for 24 h until a confluent monolayer was formed. A scratch was introduced across the cell monolayer using a 200 µL pipette tip, followed by gentle washing with PBS to remove detached cells. The initial scratch width (0 h) was recorded using an inverted fluorescence microscope. DMEM containing the LDTG A‐MN extract was then added, and the cells were incubated under the same conditions for an additional 24 h. After incubation, the scratch area was imaged again using the same microscope. Scratch closure was quantified using Image‐Pro Plus 6.0 software according to the following equation: Migration area (%) = (W_0_ – W_t_) / W_0_ × 100%, where W_0_ is the initial scratch width and W_t_ is the scratch width after 24 h.

### 4.12 Evaluation of LDTG A‐MNs in Promoting Macrophage Polarization

RAW 264.7 macrophages were cultured in DMEM containing the LDTG A‐MN extract for 24 h. Cells treated with LPS served as the control group. After incubation, the cells were collected, stained with CD86 and CD206 antibodies, and analyzed by flow cytometry (CytoFLEX S) to quantify macrophage polarization. Immunofluorescence staining was performed to visualize CD86 and CD206 expression. Additionally, the concentrations of IL‐6 and IL‐10 in the culture supernatants were measured using ELISA kits.

### 4.13 Assessment of Therapeutic Efficacy for Bacterial Infected Wounds

To evaluate the antibacterial efficacy of LDTG A‐MNs under US irradiation and the sustained release of ATRA to promote wound healing, a mouse model of infected wounds was employed. All animal experiments were performed according to the protocols approved by the Institutional Animal Care and Use Committee of Wuhan University (WP20240424). Mice were randomly divided into six groups (five mice per group): Blank, A‐MN, LD A‐MN, LDTG A‐MN, LD A‐MN + US, and LDTG A‐MN + US. MN patches were applied to the infected wounds, with a single application per treatment. For the US irradiation groups, treatment involved daily exposure to 1 MHz ultrasound (0.97 W/cm^2^, 50% duty cycle) for 10 min over three consecutive days. On the third day of treatment, wound tissue samples were collected from each group, homogenized, and analyzed for bacterial clearance using plate counting methods. Wound healing progression was documented by photographing the wounds on days 0, 3, 6, and 9. The wound area was quantified using Image‐Pro Plus 6.0 software, and the percentage of wound closure was calculated as follows: Wound area (%) = Actual wound area / Original wound area × 100%, where “Actual wound area” is the wound size at the time of measurement, and “Original wound area” is the initial wound size. Histological analysis of the healed tissue around the wound was performed at the end of the treatment period. Immunohistochemistry for IL‐6 was used to assess the expression of inflammatory markers at the wound site, and immunofluorescence staining was conducted to evaluate CD31 expression. On day 9 post‐treatment, mice were euthanized, and major organs (heart, liver, spleen, lungs, and kidneys) were harvested. The organs were fixed in 4% paraformaldehyde for 72 h, followed by H&E staining for histological analysis to assess potential systemic toxicity of the MN patches. Quantitative histological analyses were performed to objectively evaluate tissue regeneration and inflammation, including epidermal thickness, collagen deposition, and inflammatory marker expression.

### 4.14 Statistical Analysis

All the results presented in this study were means ± standard deviation (SD). Statistical analysis was conducted using a two‐sided Student's *t*‐test or one‐way analysis of variance (ANOVA), with *p* < 0.05 considered statistically significant (*p < 0.05, **p < 0.01, ***p < 0.001, and ****p < 0.0001).

## Conflicts of Interest

The authors declare no conflicts of interest.

## Supporting information




**Supporting File**: advs75589‐sup‐0001‐SuppMat.docx.

## Data Availability

The data that support the findings of this study are available from the corresponding author upon reasonable request.

## References

[1] M. Falcone , B. De Angelis , F. Pea , et al., “Challenges in the Management of Chronic Wound Infections,” Journal of Global Antimicrobial Resistance 26 (2021): 140–147, 10.1016/j.jgar.2021.05.010. 34144200

[2] S. Guo and L. A. Dipietro , “Factors Affecting Wound Healing,” Journal of Dental Research 89 (2010): 219–229, 10.1177/0022034509359125. 20139336 PMC2903966

[3] F. Ilyas , A. James , S. Khan , et al., “Multidrug‐Resistant Pathogens in Wound Infections: A Systematic Review,” Cureus 16 (2024): 58760. 10.7759/cureus.58760 PMC11111159 38779271

[4] A. Roth , A. Elkashif , V. Selyamani , et al., “Wearable and Flexible Ozone Generating System for Treatment of Infected Dermal Wounds,” Frontiers in Bioengineering and Biotechnology 8 (2020): 458. 32509746 10.3389/fbioe.2020.00458 PMC7249782

[5] A. Roth , M. K. Maruthamuthu , S. Nejati , et al., “Wearable Adjunct Ozone and Antibiotic Therapy System for Treatment of Gram‐negative Dermal Bacterial Infection,” Scientific Reports 12 (2022): 13927, 10.1038/s41598-022-17495-3. 35977975 PMC9385669

[6] A. Roth , A. Krishnakumar , R. R. McCain , et al., “Biocompatibility and Safety Assessment of Combined Topical Ozone and Antibiotics for Treatment of Infected Wounds,” ACS Biomaterials Science & Engineering 9 (2023): 3606–3617, 10.1021/acsbiomaterials.2c01548. 37235768

[7] Q. Wu , F. Zhang , X. Pan , et al., “Surface Wettability of Nanoparticle Modulated Sonothrombolysis,” Advanced Materials 33 (2021): 2007073. 10.1002/adma.202007073 33987928

[8] J. Ouyang , Z. Tang , N. Farokhzad , et al., “Ultrasound Mediated Therapy: Recent Progress and Challenges in Nanoscience,” Nano Today 35 (2020): 100949, 10.1016/j.nantod.2020.100949.

[9] Z. Li , J. Han , L. Yu , et al., “Synergistic Sonodynamic/Chemotherapeutic Suppression of Hepatocellular Carcinoma by Targeted Biodegradable Mesoporous Nanosonosensitizers,” Advanced Functional Materials 28 (2018): 1800145, 10.1002/adfm.201800145.

[10] K. M. M. Nowak , M. R. R. Schwartz , V. R. R. Breza , and R. J. J. Price , “Sonodynamic Therapy: Rapid Progress and New Opportunities for Non‐invasive Tumor Cell Killing With Sound,” Cancer Letters 532 (2022): 215592, 10.1016/j.canlet.2022.215592. 35151824 PMC8918024

[11] Y. T. Shen , S. Y. Li , X. D. Hou , et al., “Ultrasound‐Triggered Nanocomposite “Lever” Hydrogels with a Full Repair System Accelerates Diabetic Foot Ulcer Repair,” Advanced Science 12 (2025): 2500720. 40344623 10.1002/advs.202500720 PMC12199430

[12] J. N. Xu , F. S. M. Zhou , L. Y. Cao , et al., “ROS‐Balancing‐Engineered Bio‐Heterojunction Hydrogel Accelerated the Infected Bone Regeneration Based on Sono‐Chemo Dynamic Therapy,” Bioactivee Materials 52 (2025): 440–459. 10.1016/j.bioactmat.2025.05.031 PMC12206024 40585389

[13] G. R. Ma , K. Cheng , X. Wang , et al., “Dual Oxygen Supply System of Carbon Dot‐loaded Microbubbles With Acoustic Cavitation for Enhanced Sonodynamic Therapy in Diabetic Wound Healing,” Biomaterials 318 (2025): 123145, 10.1016/j.biomaterials.2025.123145. 39874643

[14] J. T. Xue , N. Q. Zhou , Y. P. Yang , et al., “Puerarin‐Loaded Ultrasound Microbubble Contrast Agent Used as Sonodynamic Therapy for Diabetic Cardiomyopathy Rats,” Colloids and Surfaces B: Biointerfaces 190 (2020): 110887. 32113166 10.1016/j.colsurfb.2020.110887

[15] H. X. Zhao , W. C. Zhou , S. J. Cao , R. Q. Guo , L. Q. Zhou , and L. Qiu , “Dual‐Enzymatic Nanocatalyst for Optimizing Hypoxia Wound Healing via Linkage‐Enhanced Sonodynamic Antimicrobial Therapy,” Small 21 (2025): 2504524. 10.1002/smll.202504524 40545835

[16] X. D. Meng , S. R. Sun , C. C. Gong , et al., “Ag‐Doped Metal–Organic Frameworks′ Heterostructure for Sonodynamic Therapy of Deep‐Seated Cancer and Bacterial Infection,” ACS Nano 17 (2023): 1174–1186, 10.1021/acsnano.2c08687. 36583572

[17] A. L. Facklam , L. R. Volpatti , and D. G. Anderson , “Biomaterials for Personalized Cell Therapy,” Advanced Materials 32 (2020): 1902005. 10.1002/adma.201902005 31495970

[18] E. Rudnitsky , A. Braiman , M. Wolfson , et al., “Stem Cell‐Derived Extracellular Vesicles as Senotherapeutics,” Ageing Research Reviews 99 (2024): 102391, 10.1016/j.arr.2024.102391. 38914266

[19] O. S. Fenton , K. N. Olafson , P. S. Pillai , M. J. Mitchell , and R. Langer , “Advances in Biomaterials for Drug Delivery,” Advanced Materials 30 (2018): 1705328. 10.1002/adma.201705328 PMC6261797 29736981

[20] J. A. Olzmann and P. Carvalho , “Dynamics and Functions of Lipid Droplets,” Nature Reviews Molecular Cell Biology 20 (2019): 137–155, 10.1038/s41580-018-0085-z. 30523332 PMC6746329

[21] A. Zadoorian , X. Du , and H. Yang , “Lipid Droplet Biogenesis and Functions in Health and Disease,” Nature Reviews Endocrinology 19 (2023): 443–459, 10.1038/s41574-023-00845-0. PMC10204695 37221402

[22] J. Yang , J. Ning , P. Sun , et al., “Fluorescent Probes—Illuminate the Interplay Network Between Lipid Droplets and Other Organelles,” Coordination Chemistry Reviews 510 (2024): 215792, 10.1016/j.ccr.2024.215792.

[23] N. L. Gluchowski , M. Becuwe , T. C. Walther , and R. V. Farese Jr. , “Lipid Droplets and Liver Disease: From Basic Biology to Clinical Implications,” Nature Reviews Gastroenterology & Hepatology 14 (2017): 343–355, 10.1038/nrgastro.2017.32. 28428634 PMC6319657

[24] M. Bosch , M. Sanchez‐Alvarez , A. Fajardo , et al., “Mammalian Lipid Droplets Are Innate Immune Hubs Integrating Cell Metabolism and Host Defense,” Science 370 (2020): 309, 10.1126/science.aay8085. 33060333

[25] D. R. Green , “Immiscible Immunity,” Science 370 (2020): 294–295, 10.1126/science.abe7891. 33060350

[26] S. Park , J. W. Ahn , Y. Jo , et al., “Label‐Free Tomographic Imaging of Lipid Droplets in Foam Cells for Machine‐Learning‐Assisted Therapeutic Evaluation of Targeted Nanodrugs,” ACS Nano 14 (2020): 1856–1865, 10.1021/acsnano.9b07993. 31909985

[27] Y. Ding , S. Zhang , L. Yang , et al., “Isolating Lipid Droplets From Multiple Species,” Nature Protocols 8 (2013): 43–51. 23222457 10.1038/nprot.2012.142

[28] T. Liang , D. Wen , G. Chen , et al., “Adipocyte‐Derived Anticancer Lipid Droplets,” Advanced Materials 33 (2021): 2100629. 10.1002/adma.202100629 33987883

[29] S. N. Gao , Y. Rao , X. W. Wang , et al., “Chlorella‐Loaded Antibacterial Microneedles for Microacupuncture Oxygen Therapy of Diabetic Bacterial Infected Wounds,” Advanced Materials 36 (2024): 2307585, 10.1002/adma.202307585. 38307004

[30] Z. S. Chen , L. Zhang , Y. Yang , et al., “Photothermal Nanozyme‐Encapsulating Microneedles for Synergistic Treatment of Infected Wounds,” Advanced Functional Materials 35 (2025): 2417415.

[31] G. H. Yang , H. W. Kang , Y. Z. Zhu , et al., “Bacteria Microenvironment‐Responsive Missile Microneedles Modulate Immunity and Penetrate Biofilm for Diabetic Wound Therapy,” Bioactive Materials 55 (2026): 426–445. 41078863 10.1016/j.bioactmat.2025.09.017 PMC12510236

[32] L. Yang , D. Zhang , W. J. Li , et al., “Biofilm Microenvironment Triggered Self‐Enhancing Photodynamic Immunomodulatory Microneedle for Diabetic Wound Therapy,” Nature Communications 14 (2023): 7658, 10.1038/s41467-023-43067-8. PMC10667311 37996471

[33] A. Krishnakumar , N. L. F. Gallina , D. Sarnaik , et al., “Microneedles for Enhanced Bacterial Pathogen Inactivation and Accelerated Wound Healing,” Advanced Materials Technologies 9 (2024): 2400219, 10.1002/admt.202400219.

[34] I. Woodhouse , S. Nejati , V. Selvamani , et al., “Flexible Microneedle Array Patch for Chronic Wound Oxygenation and Biofilm Eradication,” ACS Applied Bio Materials 4 (2021): 5405–5415. 10.1021/acsabm.1c00087 35006756

[35] C. J. Liu , K. Liu , D. Zhang , et al., “Dual‐Layer Microneedles With NO/O_2_ Releasing for Diabetic Wound Healing via Neurogenesis, Angiogenesis, and Immune Modulation,” Bioactive Materials 46 (2025): 213–228. 39802419 10.1016/j.bioactmat.2024.12.012 PMC11719290

[36] H. X. Li , X. L. Zheng , Z. K. Gao , et al., “ROS‐Responsive Core‐Shell Microneedles Based on Simultaneous Efficient Type I/II Photosensitizers for Photodynamic Against Bacterial Biofilm Infections,” Advanced Functional Materials 35 (2025): 2401477, 10.1002/adfm.202401477.

[37] J. H. Ran , Z. P. Xie , L. W. Yan , et al., “Oxygen‐Propelled Dual‐Modular Microneedles with Dopamine‐Enhanced RNA Delivery for Regulating Each Stage of Diabetic Wounds,” Small 20 (2024): 2404538. 10.1002/smll.202404538 39105463

[38] S. R. Sun , X. D. Meng , J. W. Xu , Z. Yang , X. J. Zhang , and H. F. Dong , “Ag‐Decorated Multivariate Metal‐organic Frameworks for Enhanced Sonodynamic Therapy of Bacterial Infections in Wound Healing,” Nano Today 55 (2024): 102181, 10.1016/j.nantod.2024.102181.

[39] P. Jiang , Y. N. Zeng , W. Zhang , J. P. Lei , X. Jiang , and W. Li , “Three‐in‐One Bilayer Microneedle Patch for Ultrasound‐activated Antibacterial Therapy and Immune Modulation in Wound Healing,” Journal of Controlled Release 388 (2025): 114375, 10.1016/j.jconrel.2025.114375. 41177464

[40] Y. N. Zeng , C. Y. Wang , J. P. Lei , et al., “Spatiotemporally Responsive Cascade Bilayer Microneedles Integrating Local Glucose Depletion and Sustained Nitric Oxide Release for Accelerated Diabetic Wound Healing,” Acta Pharmaceutica Sinica B 14 (2024): 5037–5052, 10.1016/j.apsb.2024.06.014. 39664438 PMC11628847

[41] T. Rhauderwiek , S. Waitschat , S. Wuttke , H. Reinsch , T. Bein , and N. Stock , “Nanoscale Synthesis of Two Porphyrin‐Based MOFs With Gallium and Indium,” Inorganic Chemistry 55 (2016): 5312–5319, 10.1021/acs.inorgchem.6b00221. 27203724

[42] P. Roingeard and R. C. N. Melo , “Lipid Droplet Hijacking by Intracellular Pathogens,” Cellular Microbiology 19 (2017): 12688, 10.1111/cmi.12688. 27794207

[43] M. R. Prausnitz , “Microneedles for Transdermal Drug Delivery,” Advanced Drug Delivery Reviews 56 (2004): 581–587, 10.1016/j.addr.2003.10.023. 15019747

[44] C. Cao , H. Zou , N. Yang , et al., “Fe _3_ O _4_ /Ag/Bi _2_ MoO _6_ Photoactivatable Nanozyme for Self‐Replenishing and Sustainable Cascaded Nanocatalytic Cancer Therapy,” Advanced Materials 33 (2021): 2106996. 10.1002/adma.202106996 34626026

