# Supplementary material for: Dual‐Stage Microneedle Patch With Lipid Droplet‐Mediated ‘Capture‐Kill’ and Sustained Release for Enhanced Wound Healing
Source: Adv Sci (Weinh). 2026 May 6;13(43):e75589. doi: 10.1002/advs.75589 (PMC13335996; doi:10.1002/advs.75589)
Supplement: Supplementary file 1 — Supporting File: advs75589‐sup‐0001‐SuppMat.docx. [file ADVS-13-e75589-s001.docx]

**Supporting Information**


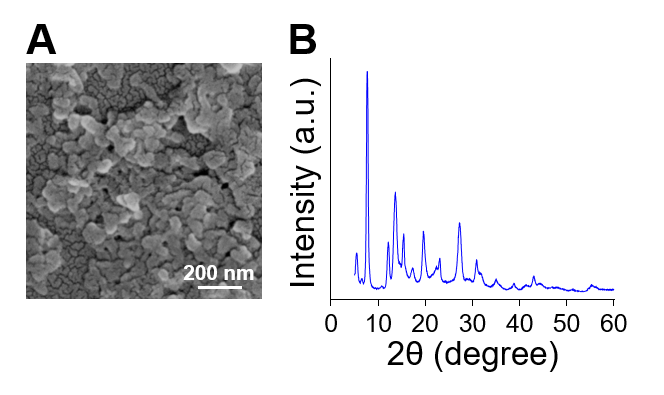


**Figure S1.** (A) SEM image, and (B) XRD of TCPPGa.


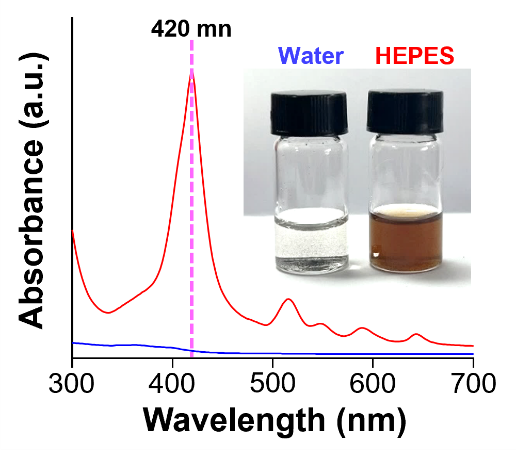


**Figure S2.** Dispersion of TCPPGa in water or HEPES and the corresponding UV-vis spectra.


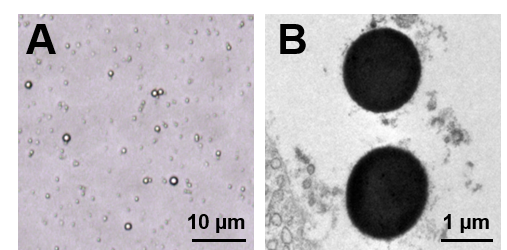


**Figure S3.** (A) Optical image and (B) TEM image of LDTG.
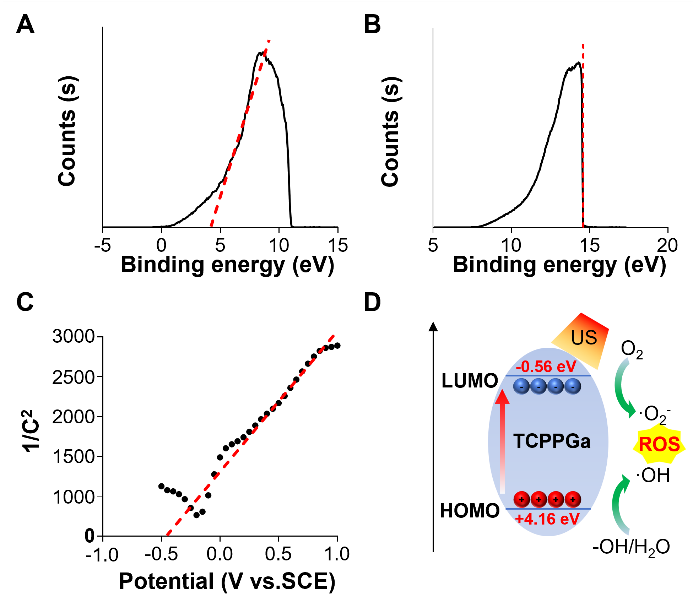


**Figure S4.** A-B) Ultraviolet photoelectron spectroscopy (UPS) spectra, and C) Mott-Schottky plots of TCPPGa. D) US-induced sonocatalytic mechanism of the TCPPGa for ROS generation.


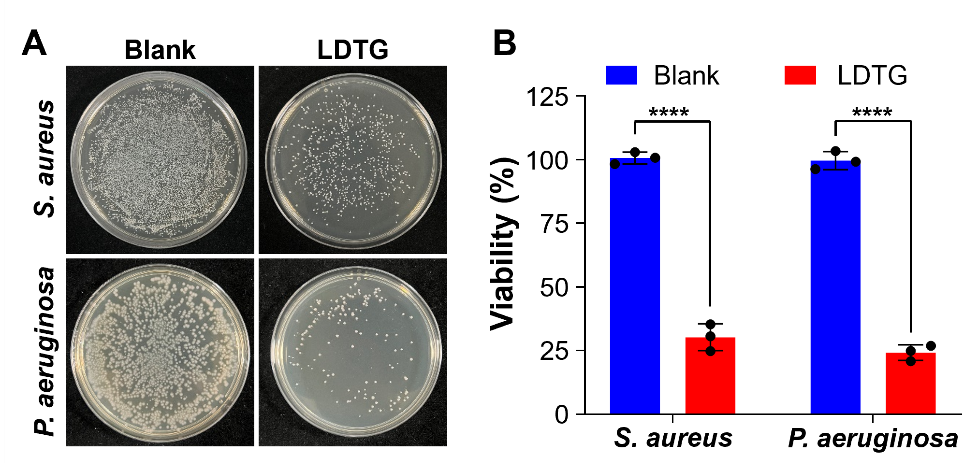


**Figure S5.** A) Representative agar plate photographs showing the bacterial viability of LDTG A-MNs on *S. aureus* and *P. aeruginosa* after bacterial capture. B) Quantitative analysis of bacterial viability following treatment with LDTG A-MNs. *****P* < 0.0001.


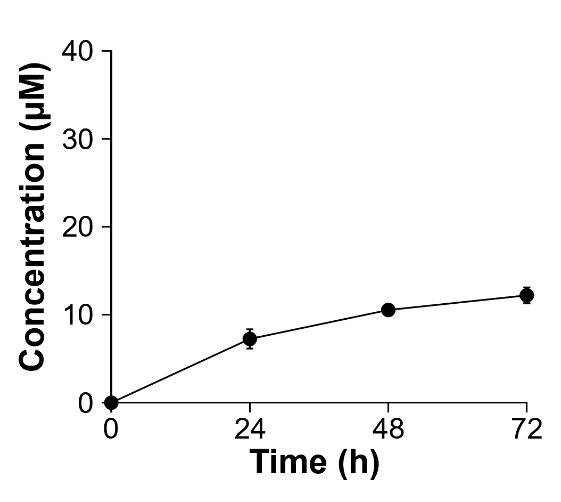


**Figure S6.** Cumulative release of Ga^3+^ from LDTG in PBS. Data are presented as mean ± SD (n = 3).


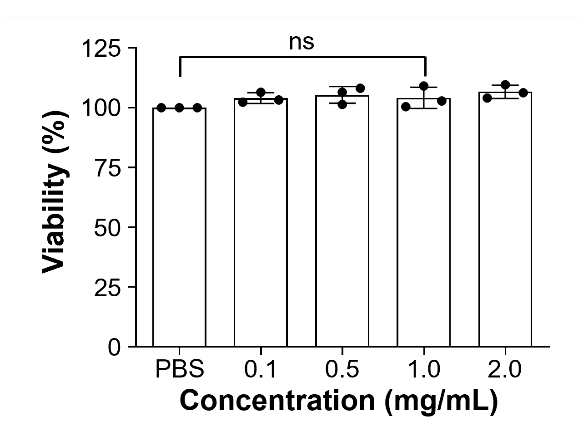


**Figure S7.** Cell viability of NIH-3T3 cells after incubation with different concentrations of LDTG for 24 h. Data are presented as mean ± SD (n = 3). The ns indicates no significance.


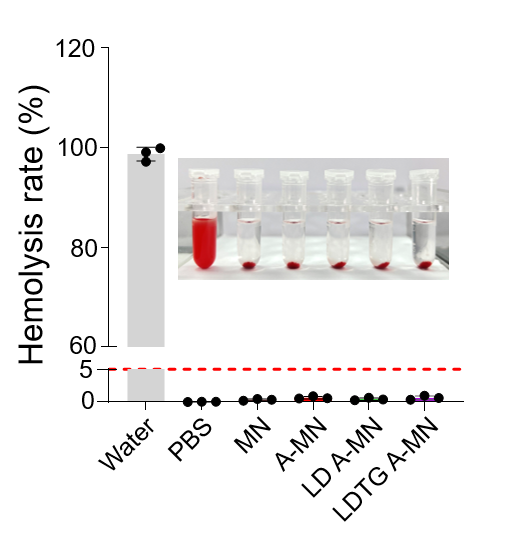


**Figure S8.** Hemolytic activity and photographs of LDTG A-MN. Data are presented as mean ± SD (n = 3).


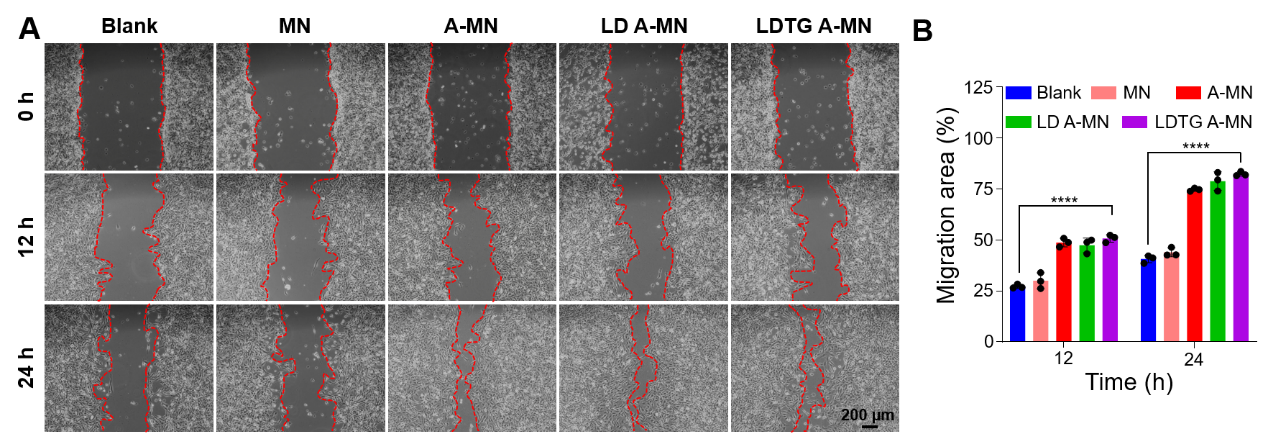


**Figure S9.** The effect of LDTG A-MN on cell migration of NIH-3T3 cells: (A) Cell scratch test and (B) migration area. Data are presented as mean ± SD (n = 3). *****P* < 0.0001.


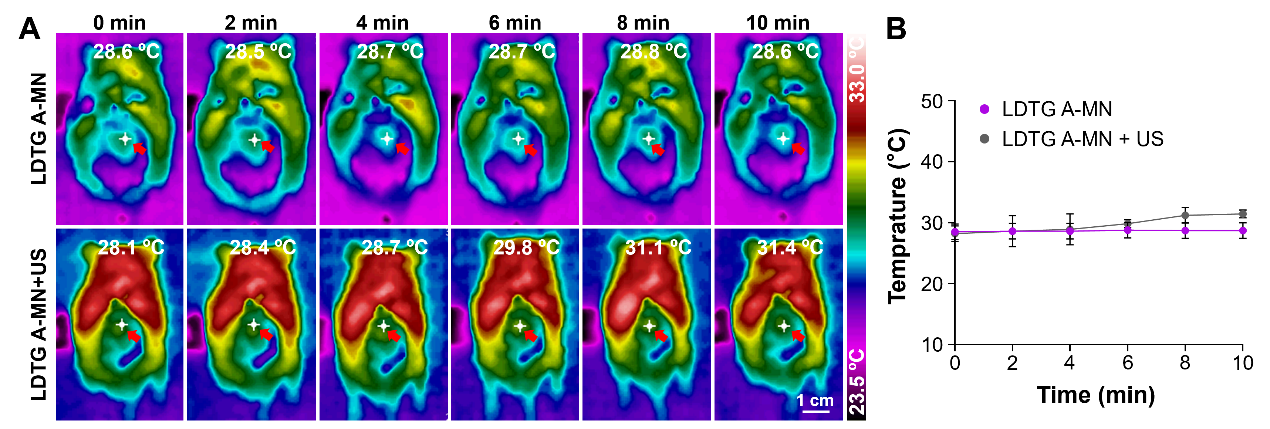


**Figure S10.** A) Thermal images and B) corresponding photothermal curves of mice treated with LDTG A-MN by US irradiation (1 MHz, 0.97 W/cm², 50% duty cycle) for 10 min. Data are presented as means ± SD (n = 3).


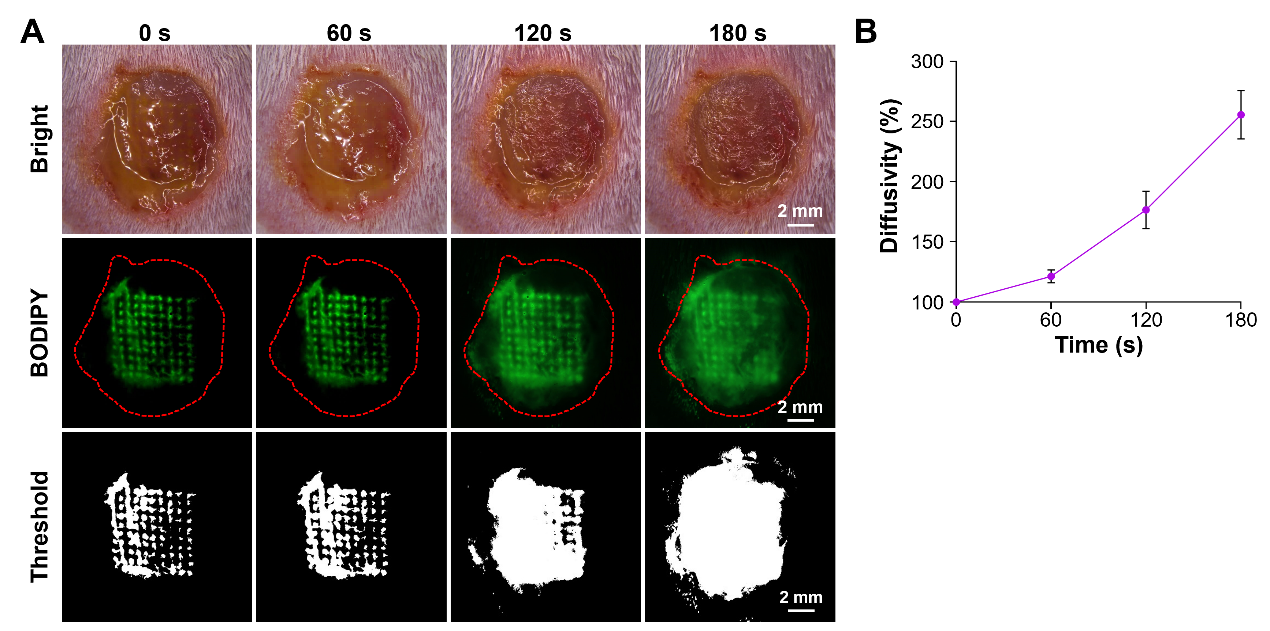


**Figure S11.** A) *In vivo* diffusion imaging and B) statistical analysis of LDTG after application of the MN patch. Data are presented as means ± SD (n = 3).


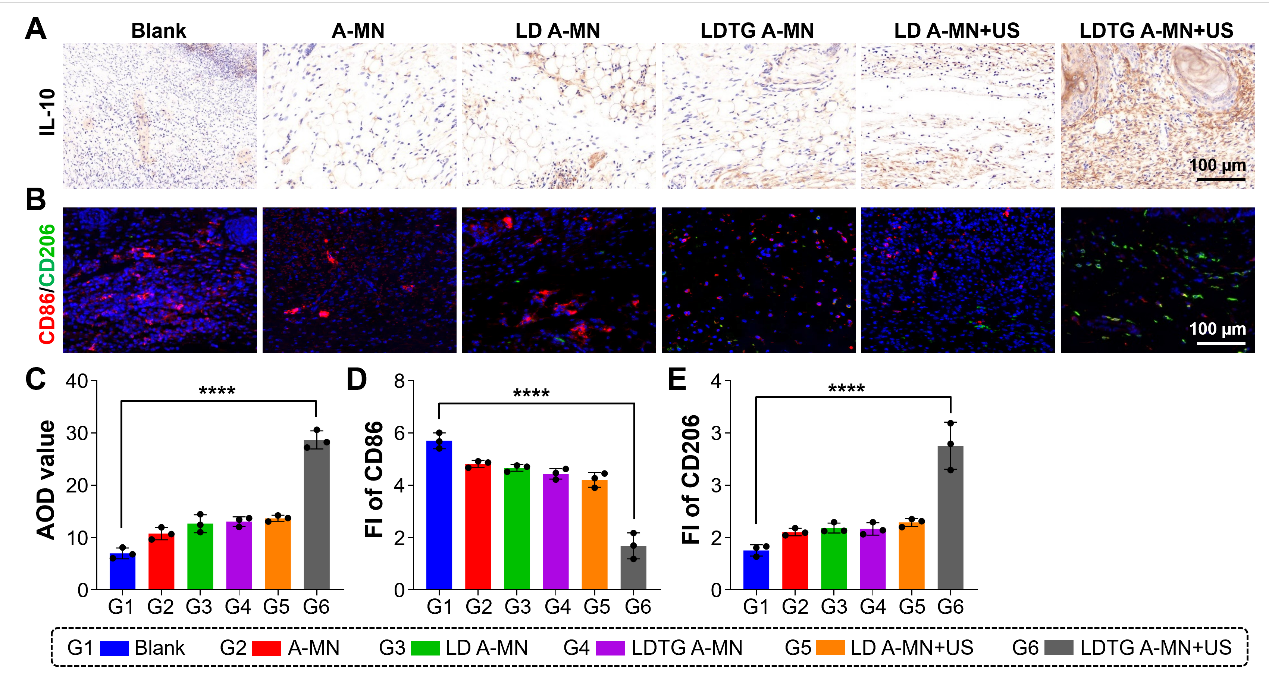


**Figure S12.** A) Immunohistochemical staining of IL-10 in wound tissues. B) Immunofluorescence staining of macrophages showing CD86 (red, M1 marker) and CD206 (green, M2 marker); nuclei were counterstained with DAPI (blue). C) Quantification of IL-10 expression using average optical density (AOD) from immunohistochemistry. D-E) Quantification of CD86 and CD206 fluorescence intensity (FI) from immunofluorescence (n = 3). *****P* < 0.0001.


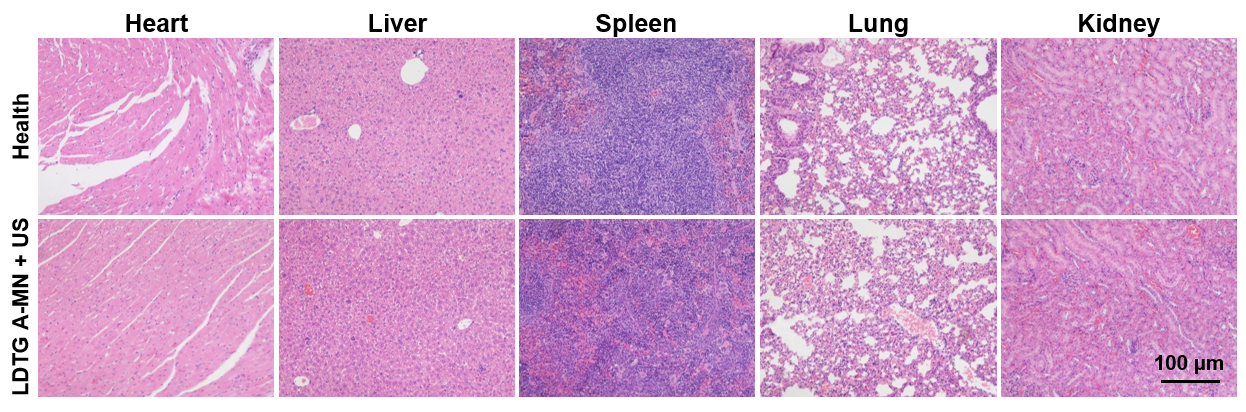


**Figure S13.** Histopathologic examination of the major organs including heart, liver, spleen, lung, and kidney from infected mice on day 9 after treatment.
